# An immunometabolic prodrug strategy overcomes DHODH inhibitor resistance in refractory melanoma

**DOI:** 10.1186/s13046-025-03566-6

**Published:** 2025-11-14

**Authors:** Yongrui Hai, Wenhui Wang, Renming Fan, Ye Chen, Junyan Zhuang, Shuo Fu, Guiquan Ding, Lei Liang, Junke Song, Gaofei Wei

**Affiliations:** 1 Institute of Medical Research, Northwestern Polytechnical University, Xi’an, 710072 China; 2 Research & Development Institute of Northwestern Polytechnical University, Shenzhen, 518057 China; 3 Institute of Materia Medica, Chinese Academy of Medical Sciences & Peking Union Medical College, Beijing, 100050 China; 4 Department of Colorectal Surgery, Fudan University Shanghai Cancer Center, Shanghai, 200032 China

**Keywords:** Immunometabolism, DHODH inhibitor resistance, STING pathway, Pyroptosis, NK cell

## Abstract

**Background:**

Metabolic reprogramming, particularly upregulated *de novo* pyrimidine biosynthesis, drives cancer progression and immune evasion. Dihydroorotate dehydrogenase (DHODH), a key enzyme in this pathway, is a promising therapeutic target, but its inhibitors often face resistance in immune-refractory melanoma, linked to low basal stimulator of interferon genes (STING) expression.

**Methods:**

To overcome this limitation, we designed H62, a tumor-selective prodrug conjugating the DHODH inhibitor EA6 with the STING agonist MSA-2 via a cathepsin B-cleavable linker. Mechanistic studies evaluated mitochondrial disruption, pyroptosis (caspase-3/GSDME), and STING-mediated interferon signaling, alongside natural killer (NK) cell recruitment. Efficacy was tested in multiple melanoma models, including standard and neoadjuvant settings.

**Results:**

H62 synergistically induced mitochondrial dysfunction and pyroptosis while activating STING/type I interferon responses, enhancing NK cell cytotoxicity. In melanoma models, it significantly suppressed tumor growth, reduced postoperative recurrence, and improved survival.

**Conclusions:**

This dual-targeting strategy overcomes DHODH inhibitor resistance by coupling metabolic interference with innate immune activation, offering translational potential for melanoma and other treatment-resistant cancers.

**Supplementary Information:**

The online version contains supplementary material available at 10.1186/s13046-025-03566-6.

## Introduction

Metabolic reprogramming is widely recognized as a hallmark of cancer, supporting not only sustained proliferation but also tumor initiation, immune evasion, and metastatic dissemination [1, 2]. Among the various rewired metabolic pathways, *de novo* pyrimidine biosynthesis has emerged as a key driver in meeting the anabolic and energetic requirements of rapidly dividing tumor cells [3]. Recent evidence indicates that beyond supporting proliferation, dysregulated pyrimidine metabolism also contributes to the formation of an immunosuppressive tumor microenvironment (TME), thereby promoting immune escape [4]. As a central metabolic organelle, mitochondria integrate redox control and biosynthetic functions; within this context, dihydroorotate dehydrogenase (DHODH)—the only mitochondrial enzyme in the pyrimidine biosynthetic pathway—has gained increasing attention for its dual role in nucleotide metabolism and cancer progression [5–7].

Beyond its canonical metabolic function, DHODH has also been implicated in tumor–immune crosstalk. Inhibition of DHODH has been shown to suppress the proliferation of regulatory T cells [8], impair the differentiation of myeloid-derived suppressor cells (MDSCs) [9], and promote phagocytic activity in macrophages—collectively contributing to a reversal of immunosuppression [10]. On the other hand, recent studies suggest that pyrimidine metabolites released from macrophages, may counteract the efficacy of certain chemotherapeutic agents (e.g., gemcitabine in pancreatic cancer), underscoring the complexity of this metabolic-immune interface [11]. In our previous work, we found that the DHODH inhibitor brequinar induces mitochondrial oxidative stress and pyroptosis in melanoma cells, accompanied by enhanced infiltration of natural killer (NK) cells and improved antitumor immunity. Based on this, we developed a more potent DHODH inhibitor, EA6, which demonstrated improved cytotoxicity and greater capacity to recruit NK cells [12]. However, while DHODH-targeted agents show promise in preclinical models, their efficacy in solid tumors remains limited, largely due to intrinsic or adaptive resistance mechanisms [13, 14]. This presents a major unresolved challenge: how to overcome both metabolic and immunological barriers that limit the therapeutic benefit of DHODH inhibition, especially in immune-cold or treatment-resistant melanoma subtypes.

Recent studies have highlighted the innate immune cGAS–STING signaling axis as a promising target for cancer immunotherapy [15, 16]. Activation of STING can initiate robust production of type I interferons (IFN-I) and inflammatory cytokines, which contribute to immune cell recruitment and activation within the TME [17–21]. Notably, IFN-I has been shown to directly enhance NK cell cytotoxic function and augment antitumor immune responses [22–24]. These findings led us to hypothesize that pharmacologic activation of the STING pathway could synergize with DHODH inhibition to boost NK cell activation and function, thereby sensitizing resistant melanoma cells to metabolic therapy.

**Scheme 1:**
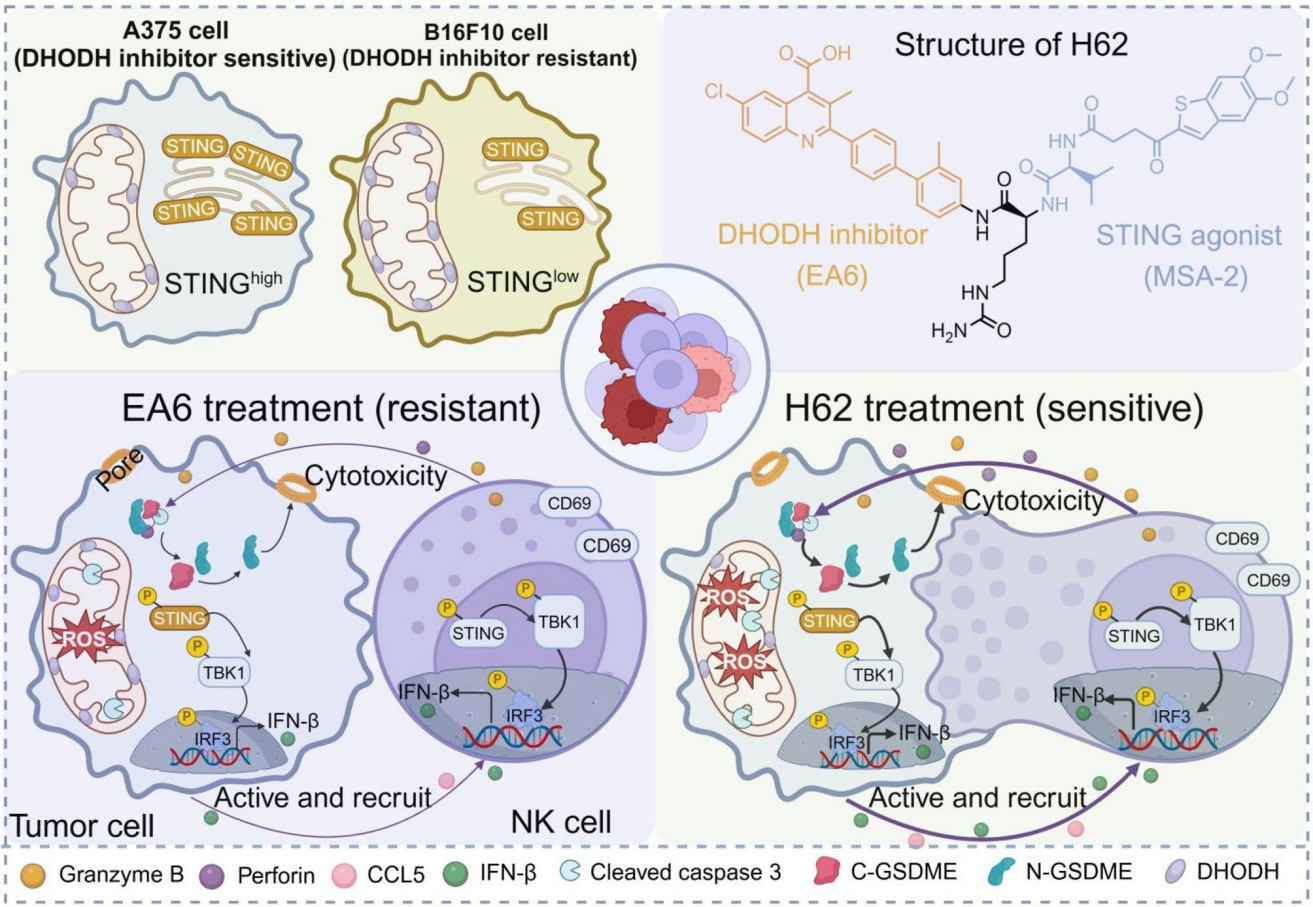
H62 enhances tumor sensitivity to DHODH inhibitors via activating the STING signaling pathway and amplifies NK cell-mediated antitumor immunotherapy. Top-left: A375 cells with high STING expression are sensitive to DHODH inhibition, whereas B16F10 cells with low STING expression exhibit resistance. Top-right: Structure of H62, a cathepsin B–cleavable prodrug linking DHODH inhibitor EA6 and STING agonist MSA-2 for tumor-selective dual activation. Bottom-left: In STING^low^ B16F10 cells, EA6 monotherapy fails to overcome DHODH inhibitor resistance, resulting in impaired pyroptosis and insufficient NK cell activation. Bottom-right: Prodrug H62 reverses DHODH inhibitor resistance by simultaneously inducing mitochondrial pyroptosis and activating STING signaling, thereby enhancing NK cell recruitment and cytotoxicity in melanoma. This figure was created using BioRender

To explore this, we rationally designed and synthesized a tumor-selective prodrug, H62, by conjugating our potent DHODH inhibitor EA6 with the validated STING agonist MSA-2 (MSA) via a cathepsin B (CTSB)-cleavable linker. This design enables the selective and coordinated release of both active agents within the protease-rich tumor microenvironment. Mechanistically, H62 was shown to induce mitochondrial dysfunction and robust pyroptosis, thereby enhancing NK cell recruitment. Importantly, activation of the STING pathway by the released MSA potentiated NK cell cytotoxicity, increasing IFN-I production and the secretion of key effector molecules such as granzyme B and perforin. Through these synergistic mechanisms, H62 effectively overcame intrinsic melanoma resistance to DHODH inhibition (Scheme 1). In both standard and neoadjuvant melanoma models, H62 significantly suppressed tumor growth, reduced recurrence, and extended survival, underscoring its potential for clinical translation.

In conclusion, our study introduces a rationally designed dual-functional prodrug that integrates pyrimidine metabolism inhibition with STING pathway activation to overcome resistance to DHODH-targeted therapy. This approach highlights a new direction for the development of immunometabolic strategies in treating melanoma and other immunotherapy-refractory malignancies.

## Methods

### Synthesis and characterization of H62

#### Intermediate 11 − 3

5-Fluoroisatin (825 mg, 4.996 mmol) and potassium hydroxide (1.683 g, 29.995 mmol) were dissolved in a mixture of 20 mL ethanol and 10 mL water, heated externally to 100 °C with stirring for 30 min, and then 4’-bromopropiophenone (1.278 g, 5.998 mmol) was added. The mixture was heated to reflux overnight, and the reaction was monitored by TLC. After the reaction was complete, the reaction mixture was diluted with ethyl acetate (200 mL), washed with saturated ammonium chloride aqueous solution (30 mL×3), and the layers were separated. It was then washed with 30 mL of water, the layers were separated again, and the organic layers were combined. The solution was dried over anhydrous sodium sulfate, filtered under reduced pressure, and the filtrate was concentrated under reduced pressure. The remaining residue was mixed with silica gel and further purified with silica gel column chromatography, yielding intermediate 11 − 3, white solid,1.467 g, 78.0%. ^1^H NMR (500 MHz, DMSO-d6) δ 7.95–7.88 (m, 2 H), 7.68 (d, J = 8.0 Hz, 2 H), 7.61 (dd, J = 8.9, 2.5 Hz, 1H), 7.52 (d, J = 8.1 Hz, 2 H), 2.30 (s, 3 H). ^13^C NMR (126 MHz, DMSO) δ 199.68, 159.46, 144.36, 140.25, 135.57, 131.74, 131.14, 130.96, 130.65, 129.86, 129.75, 128.67, 127.05, 125.67, 124.48, 121.49, 17.51.

#### Intermediate 11 − 4

Intermediate 11 − 3 (376 mg, 1.003 mmol), 4-amino-2-methylphenylboronic acid pinacol ester (350 mg, 1.501 mmol), and sodium carbonate (2.500 mmol) were dissolved in a mixture of 9 mL of 1,4-dioxane and 3 mL of water. Under nitrogen protection, tetrakis(triphenylphosphine)palladium(0) (57 mg, 0.049 mmol) was added, and the mixture was heated externally to 180 °C and reacted overnight. The reaction was monitored by TLC. After the reaction was complete, the incompletely reacted palladium catalyst and metallic palladium were filtered through celite. The filtrate was diluted with 70 mL of ethyl acetate, washed with saturated ammonium chloride aqueous solution (20 mL × 3), and the layers were separated. It was then washed with 20 mL of water, the layers were separated again, and the organic layers were combined. The solution was dried over anhydrous sodium sulfate, filtered under reduced pressure, and the filtrate was concentrated under reduced pressure. The remaining residue was mixed with silica gel and purified with silica gel column chromatography, yielding intermediate 11 − 4, white solid, 344 mg, 85.3%. ^1^H NMR (500 MHz, DMF-d7) δ 8.18 (s, 1H), 8.02 (d, J = 2.4 Hz, 1H), 7.89 (dt, J = 8.9, 2.7 Hz, 1H), 7.76 (d, J = 7.9 Hz, 2 H), 7.56 (d, J = 7.8 Hz, 2 H), 7.15 (d, J = 8.0 Hz, 1H), 6.69 (d, J = 12.0 Hz, 2 H), 2.58 (s, 3 H), 2.38 (s, 3 H). ^13^C NMR (126 MHz, DMSO-d6) δ 169.91, 161.05, 148.48, 144.68, 142.49, 138.04, 135.50, 131.59, 131.48, 130.83, 129.91, 129.37, 129.22, 129.20, 128.98, 128.78, 128.56, 124.66, 124.41, 124.04, 116.23, 112.41, 21.03, 18.21.

#### Intermediate E-02

Intermediate 11 − 4 (200 mg, 0.496 mmol), HATU (377 mg, 0.992 mmol) and (S)−2-((S)−2-((tert-butoxycarbonyl)amino)−3-methylbutanoylamino)−5-ureidopentanoic acid (278 mg, 0.744 mmol) were dissolved in 8 mL of dry dichloromethane. N, N-Diisopropylethylamine (192 mg, 1.488 mmol) was then added, and the reaction was allowed to proceed under nitrogen protection for 16 h. The reaction was monitored by TLC. After the reaction was complete, the reaction mixture was diluted with 50 mL of dichloromethane, washed with saturated ammonium chloride aqueous solution (10 mL×3), and the layers were separated. It was then washed with 10 mL of water, the layers were separated again, and the organic layers were combined. The solution was dried over anhydrous sodium sulfate, filtered under reduced pressure, and the filtrate was concentrated under reduced pressure. The remaining residue was mixed with silica gel and purified by silica gel column chromatography, yielding intermediate E-02, white solid, 272 mg, 72.3%. ^1^H NMR (500 MHz, DMSO-d6) δ 10.08 (s, 1H), 8.71 (ddd, J = 4.6, 3.3, 1.6 Hz, 1H), 8.48 (dq, J = 8.4, 1.6 Hz, 1H), 8.03 (d, J = 8.9 Hz, 2 H), 7.81 (d, J = 2.3 Hz, 1H), 7.76 (dd, J = 8.8, 2.3 Hz, 1H), 7.66 (d, J = 7.8 Hz, 2 H), 7.59–7.54 (m, 2 H), 7.47–7.45 (m, 2 H), 7.24 (d, J = 8.9 Hz, 1H), 6.03 (d, J = 8.0 Hz, 1H), 5.47–5.40 (m, 2 H), 4.47 (q, J = 7.4 Hz, 1H), 3.85 (dd, J = 9.0, 6.7 Hz, 1H), 3.03 (q, J = 5.4, 4.1 Hz, 1H), 2.96 (dq, J = 13.1, 6.5 Hz, 2 H), 2.43 (s, 3 H), 2.29 (s, 3 H), 1.98 (q, J = 6.8 Hz, 1H), 1.75–1.68 (m, 1H), 1.66–1.58 (m, 1H), 1.47 (s, 1H), 1.39 (s, 6 H), 1.38 (s, 4 H), 0.85 (dd, J = 20.7, 6.7 Hz, 7 H). ^13^C NMR (126 MHz, CD3OD_SPE) δ 170.29, 167.92, 158.29, 145.42, 144.24, 141.09, 138.86, 135.94, 135.80, 134.42, 134.34, 132.60, 128.89, 126.71, 126.48, 126.35, 125.89(2 C), 125.45, 125.41(2 C), 123.95, 121.57, 121.31, 120.59, 116.55, 76.34, 62.24, 59.86, 35.73, 28.31, 27.47, 27.28, 25.70, 24.33, 24.29, 16.45, 15.41, 14.22, 13.72.

#### Intermediate E-03

Intermediate E-02 (240 g, 0.316 mmol) was dissolved in 4.8 mL of anhydrous methanol, and the temperature was controlled to around 0 °C using ice water. A solution of hydrochloric acid in 1,4-dioxane (2.4 mL) was then added dropwise. After the reaction temperature returned to room temperature, the reaction was allowed to proceed for 2 h. The reaction was monitored by TLC. After the reaction was complete, the reaction mixture was concentrated under reduced pressure, yielding intermediate E-03, white solid, 190 mg, 91.3%.

#### Compound H62

E-03 (150 mg, 0.228 mmol), MSA (101 mg, 0.342 mmol), and HATU (173 mg, 0.456 mmol) were dissolved in 6 mL of dry dichloromethane. N, N-Diisopropylethylamine (88 mg, 0.684 mmol) was then added, and the reaction was allowed to proceed overnight under nitrogen protection. The reaction was monitored by TLC. After the reaction was complete, the reaction mixture was diluted with 50 mL of dichloromethane, washed with saturated ammonium chloride aqueous solution (10 mL×3), and the layers were separated. It was then washed with water (10 mL), the layers were separated again, and the organic layers were combined. The solution was dried over anhydrous sodium sulfate, filtered under reduced pressure, and the filtrate was concentrated under reduced pressure. The remaining residue was mixed with silica gel and purified by silica gel column chromatography, yielding H62, white solid, 272 g, 72.3%. ^1^H NMR (500 MHz, DMSO-d6) δ 9.93–9.81 (m, 1H), 8.27–8.06 (m, 3 H), 7.98–7.88 (m, 2 H), 7.60 (tdd, J = 25.2, 12.9, 6.1 Hz, 6 H), 7.45–7.29 (m, 3 H), 7.13 (ddd, J = 15.9, 8.1, 2.4 Hz, 1H), 6.29–6.11 (m, 1H), 5.51 (t, J = 14.9 Hz, 2 H), 4.40 (dq, J = 13.3, 6.4, 5.4 Hz, 1H), 4.20 (t, J = 7.2 Hz, 1H), 3.81 (dd, J = 28.0, 7.5 Hz, 6 H), 3.02 (dt, J = 23.3, 6.9 Hz, 2 H), 2.64 (t, J = 7.2 Hz, 1H), 2.44–2.32 (m, 3 H), 2.21 (t, J = 3.7 Hz, 3 H), 2.01 (dq, J = 42.7, 6.9 Hz, 2 H), 1.86–1.64 (m, 2 H), 1.53–1.38 (m, 2 H), 1.30–1.20 (m, 2 H), 0.90 (t, J = 6.9 Hz, 6 H). ^13^C NMR (126 MHz, CD3OD_SPE) δ 194.81, 175.66, 174.86, 152.66, 150.31, 149.81, 145.52, 143.10, 142.01, 140.10, 138.65, 136.78, 135.55, 134.29, 133.29, 131.39, 131.05, 130.89, 130.79, 130.69, 130.56, 130.19, 129.72, 128.95, 128.58, 126.50, 126.25, 125.99, 125.70, 125.00, 123.38, 119.27, 107.65, 104.95, 68.74, 64.25, 56.67, 56.53, 55.25, 33.05, 32.85, 30.75, 30.72, 25.06, 23.71, 20.88, 19.86, 19.31, 18.13. HRMS (ESI) calcd for C_49_H_51_ClN_6_O_9_S, [M-H]^−^ 935.3205, found 935.3170.

### Cell culture

A375 (human melanoma cells, Procell, CL-0014, RRID: CVCL_0132), B16F10 (murine melanoma cells, Procell, CL-0319, RRID: CVCL_0159), HUVEC (human umbilical vein endothelial cells, Procell, CP-H082). They were cultured in Dulbecco’s Modified Eagle’s Medium (DMEM) or RPMI − 1640 medium supplemented with 10% fetal bovine serum (ExCell Bio, FSP500), penicillin (100 U/mL), and streptomycin (100 µg/mL), respectively, at 37 ℃ with 5% CO₂. NK92 (human natural killer cells, Procell, CL-0530, RRID: CVCL_2142), which were derived from peripheral blood mononuclear cells of a 50 - year - old white male with aggressive non - Hodgkin’s lymphoma, were cultured in a specific medium (Procell, CM-0530) supplemented with 0.2% interleukin − 2 (IL − 2). In this study, the cell lines involved were confirmed to be correct and free of contamination after short tandem repeat (STR) analysis and quality inspection.

### Animals

Female C57BL/6 mice aged 6–8 weeks were purchased from Keaoke Biotechnology Co., Ltd. The mice were housed in pathogen-free environment, with temperature maintained 21–23 ℃ and they were provided with ad libitum access to a standard diet and water throughout the study period. All procedures conducted on animals complied with the ARRIVE guidelines (EU Directive 2010/63/EU for animal experiments). All the experiments involving animals were performed according to protocols approved by the Institutional Animal Care and Use Committee of Northwestern Polytechnical University (202301196).

### Drug release assay

Incubate the prodrug H62 with 5U of cathepsin B (Sino Biological, 50084 - M08H) in the reaction buffer (0.1 M PBS, 15 mM ethylene diamine tetraacetic acid, 30 mM dithiothreitol) at 37 °C. At the set time points (0, 2, 6, 12, 24 h), take 50 µL of samples, immediately add 200 µL of methanol to terminate the reaction and precipitate the proteins. Vortex for 3 min, centrifuge at 13 000 rpm for 10 min, then take the supernatant and determine the concentration of the active drug in the supernatant by HPLC.

When measuring the release of H62 in cells, incubate the cells with the H62 drug solution for 2, 6, 12, and 24 h respectively. Collect the B16F10 cells together with the drug - containing medium. After sonicating the B16F10 cells, freeze - dry them, re - dissolve them with a small amount of water, add methanol, vortex and centrifuge to take the supernatant, and measure the drug concentration with an ultraviolet absorption meter.

### Live and dead cells staining

Cells were seeded in 12-well plates until reaching a confluency of approximately 70% and treated with DMSO, EA6, MSA and H62 solutions for 24 h, following treatment, cells were washed with PBS for three times and then stained with cAM/PI according to protocols and result were analysis by fluorescence microscope.

### Cytotoxicity assay

Cells were seeded in 96-well plates and treated with various compounds solutions at different concentrations for 24 h. Following this incubation period, methyl thiazolyl tetrazolium (MTT) was added into cell culture plates at a concentration of 0.5 µg/mL for 4 h. Subsequently, dimethyl sulfoxide (DMSO) was added for a duration of 15 min and cytotoxicity was detected by using an enzyme-linked immunosorbent assay (Tecan-Tecan Spark) at an absorbance wavelength of 490 nm.

### Cell morphology observation

A375 and B16F10 cells were grown in 12-well plates and accepted various administration for 24 h, respectively. Following treatment the different experimental group cells morphology were observed by microscope.

### Colony formation assay

Cells were seeded in 12-well plates at a density of 1 × 10^3^/well and administrated for 24 h. After 24 h, replace the different compounds containing medium with fresh medium and then cells were cultured for 7 days. Subsequently, cells were stained with Giemsa staining solution (Beyotime, C0133) for 15 min and washed with PBS for three times, take photos after drying.

### Western blot

Cell were seeded in 6-well plates and treated with indicated compounds solutions for 24 h, after the indicated incubation duration, protein lysates were obtained using RIPA buffer supplemented with phosphatase enzyme A inhibitor and phenylmethylsulfonyl fluoride (PMSF). The proteins were then separated using SDS-PAGE and subsequently transferred to a PVDF membrane. Following the transfer, the membrane were blocked by 5% non-fat milk at room temperature for 1 h. Next, incubated with primary antibodies overnight at 4 ℃, followed by three washes with TBST. Secondary antibodies were applied for 2 h at room temperature, followed by additional three washes with TBST. Eventually, the protein bands were visualized and obtained by a Chemiluminescence Imaging System (VILBER-VILBER FUSION FX6. EDGE). The primary antibody used in this study including DHODH (Proteintech, 14877, RRID: AB_2091723), Caspase 3 (Cell Signaling Technology, 14220, RRID: AB_2798429), Cleaved caspase 3 (Cell Signaling Technology, 9664, RRID: AB_2070042), GSDME (Abcam, 215191, RRID: AB_2737000), STING (Cell Signaling Technology, 50494, RRID: AB_2799375), p-STING (Cell Signaling Technology, 72971, RRID: AB_2799831), TBK1 (Cell Signaling Technology, 3504, RRID: AB_2255663), p-TBK1 (Cell Signaling Technology, 5483, RRID: AB_10693472), IRF3 (Cell Signaling Technology, 4302, RRID: AB_1904036), p-IRF3 (Cell Signaling Technology, 29047, RRID: AB_2773013), Vinculin (Abcam, 129002, RRID: AB_11144129), The secondary antibodies used was goat anti-rabbit IgG H&L (HRP) (Abcam, 205718, RRID: AB_2819160).

### Extract primary NK cells

After euthanizing the mice, the spleen was removed and ground before passing through a 70 mesh sieve. Red blood cell lysis buffer was added to avoid light and the precipitate was collected by centrifugation. Extract NK cells using an NK cell isolation kit (Biolegend, 480050) on ice, resuspend the cells in MojoSort™ buffer, mix well with Biotin-Antibody Cocktail, and then incubate on ice for 15 min. Vortex, add Streptavidin coupled nanomagnetic beads, mix well, and incubate on ice for 15 min. Next, add buffer to resuspend the cells, centrifuge, discard the supernatant, add buffer again, incubate the flow cytometer in a magnetic pole for 5 min, collect the liquid, repeat sorting twice, and collect all cells for later use. NK cells were cultured in MEM medium supplemented with IL-2 (1000 IU/mL) and activated NK cells were obtained after 5 days.

### Mitochondrial function analysis

A375 and B16F10 cells were seeded in 12-well plates and treated with DMSO, EA6, MSA, H62 at indicated concentrations for 24 h. After incubation, cells were washed with PBS for three times. To add CellRox (Invitrogen, C10448) as a ROS indicator and JC-1 (Beyotime, C2003) was employed as a mitochondrial membrane potential probe following the manufacture’s instructions.

### Inhibitors treatment

Ferroptosis inhibitor treatment: Pre-treat the cells with 20 µM Fer-1 (S7243, SelleckChem) for 30 min and then add different concentrations of BRQ for 48 h.

### Enzyme linked immunosorbent assay (ELISA)

Cytokines secreted from cells, tumor tissues and serum were detected using ELISA kits. Cytokines in cell culture supernatant and serum were directly collected and cytokines in tumor tissues were extracted using ELISA tissue lysate solution at 4℃ following by manufacture’s instructions. ELISA assay kits used in this study including IFN-β (R&D SYSTEMS, DY8234-05) IFN-γ (Invitrogen, 88–7314-88), CCL5 (Invitrogen, 88–56009-88), TNF-α (Invitrogen, 88–7324-88) and IL-1β (Invitrogen, 88–7013-22).

### Cell transfection

Small interference RNAs (siRNAs) targeting the mRNA sequence of DHODH (siDHODH) and a negative control siRNA (siNC) were purchased from Tsingke Biotechnology Co.,Ltd. (Beijing, China). Three various siDHODH duplexes were detected and siDHODH sequences were shown in Table S1. Both A375 and B16F10 cells were transfected 100 nM siDHODH using Lipofectamine 2000 (Invitrogen, 11668-019) for 48 h.

Short-hairpin RNAs (shRNAs) targeting the mRNA sequence of STING (shSTING) and a negative control shRNA (shNC) were generated from Tsingke Biotechnology Co.,Ltd., and transfected into B16F10 cells following the manufacture’s instructions. The efficacy of STING knockdown was verified using western blot. The shSTING sequences were shown in Table S2.

### Immunological synapse formation assay

Carboxyfluorescein diacetate succinimidyl ester (CFSE-SE) (Yeasen, 40715ES25) labeled NK cells and Celltracker CM-Dil (Yeasen, 40718ES60) labeled B16F10 cells in different treatment groups, co-culture B16F10 and NK cells for 6 h after staining and then immunological synapse formation were observed by confocal laser scanning microscope (CLSM, OLYMPUS FV3000).

### Flow cytometry analysis

After various treatments, tumor tissues, spleens and TDLNs were dissected after mice were euthanized and then digestion or homogenization to obtain single cells. Next, cells were stained with specific antibody followed by manufacture’s protocols and analyzed by flow cytometry. Flow Jo_ V10 software was utilized for data analysis and interpretation. Similarly, cells treated with different compounds were collected and stained with specific antibody to further perform flow cytometry analysis. The antibody used in this study including CD3 (Cell Signaling Technology, 46233, RRID: AB_2799297), CD45 (Biolegend, 103132, RRID: AB_893340), NK1.1 (Biolegend, 108707, RRID: AB_313394), CD69 (Biolegend, 96127), Granzyme B (Biolegend, 372222, RRID: AB_2728389), Perforin (Biolegend, 154308).

### Anti-tumor efficacy in vivo

To evaluate the anti-tumor efficacy in vivo, a subcutaneous B16F10-bearing tumor model were established by injecting 5 × 10^5^ cells per mouse into right flank on day 0. Mice were randomly divided into four groups when tumors volume reached approximately 50 mm^3^ and intraperitoneal injection administration. If necessary, all experimental animals will be anesthetized with Isoflurane for subsequent experiments. The selection of concentrations, doses, route and frequency of compound administration were determined through pre-experiments. The mice body weight and tumors volume were measured and recorded every two days during experiment. At the end of experiment, the tumors of each group were dissected for immunohistochemitry, immunofluorescence staining to detect the related indexes. Moreover, the major organ and blood were collected for H&E staining and blood routine to assessed side effect of compounds. The tumor volume (V) were calculated using the following formula, V = a × b^2^/2, where ‘a’ and ‘b’ represent longest and shortest dimension of the tumors, respectively.

### Mechanism exploration in vivo of H62

In the uridine supplement experiment conducted in vivo, 50 µL of 0.2 µM uridine was injected directly into the tumors. To investigate the role of STING in tumor growth, a STING knockdown B16F10 tumor-bearing model was established by injecting STING knockdown B16F10 cells into mice.

For the NK cell depletion experiment, 250 µg of anti-NK1.1 (BioXCell, BE0036, RRID: AB_1107737) or IgG2a (BioXCell, BE0085, RRID: AB_1107771) antibodies were diluted in InVivoPure pH 7.0 dilution buffer and injected intraperitoneally into the mice on days 6, 9, 12, and 15. To confirm the depletion of NK cells, spleens were collected 24 h after the first injection of anti-NK1.1 antibody, and flow cytometry analysis was performed. This experiment aimed to assess the impact of NK cell depletion on tumor growth in the context of the STING knockdown B16F10 tumor-bearing model.

In the experiment of combined use of immune checkpoint inhibitors, anti-mouse PD − 1 (BioXCell, BE0273, RRID: AB_2687796) was diluted to 200 µg with InVivoPure pH 7.0 dilution buffer, and each mouse was intraperitoneally injected with 200 µg of PD − 1 on days 7, 10, 13, and 16 for treatment.

### Statistical analysis

The data analysis were performed using GraphPad prism 9.5.0 software and results were presented as mean ± standard deviation (SD). Each experiment were repeated at least three times to ensure the reliability of the data. Comparisons between two groups were evaluated using an unpaired t-test, while comparisons involving multiple groups were assessed through one-way ANOVA. A significance threshold of *P* < 0.05 was considered statistically significant, whereas *P* > 0.05 was regarded as not statistically significant.

## Results

### STING expression modulates melanoma sensitivity to DHODH Inhibition

**Fig. 1:**
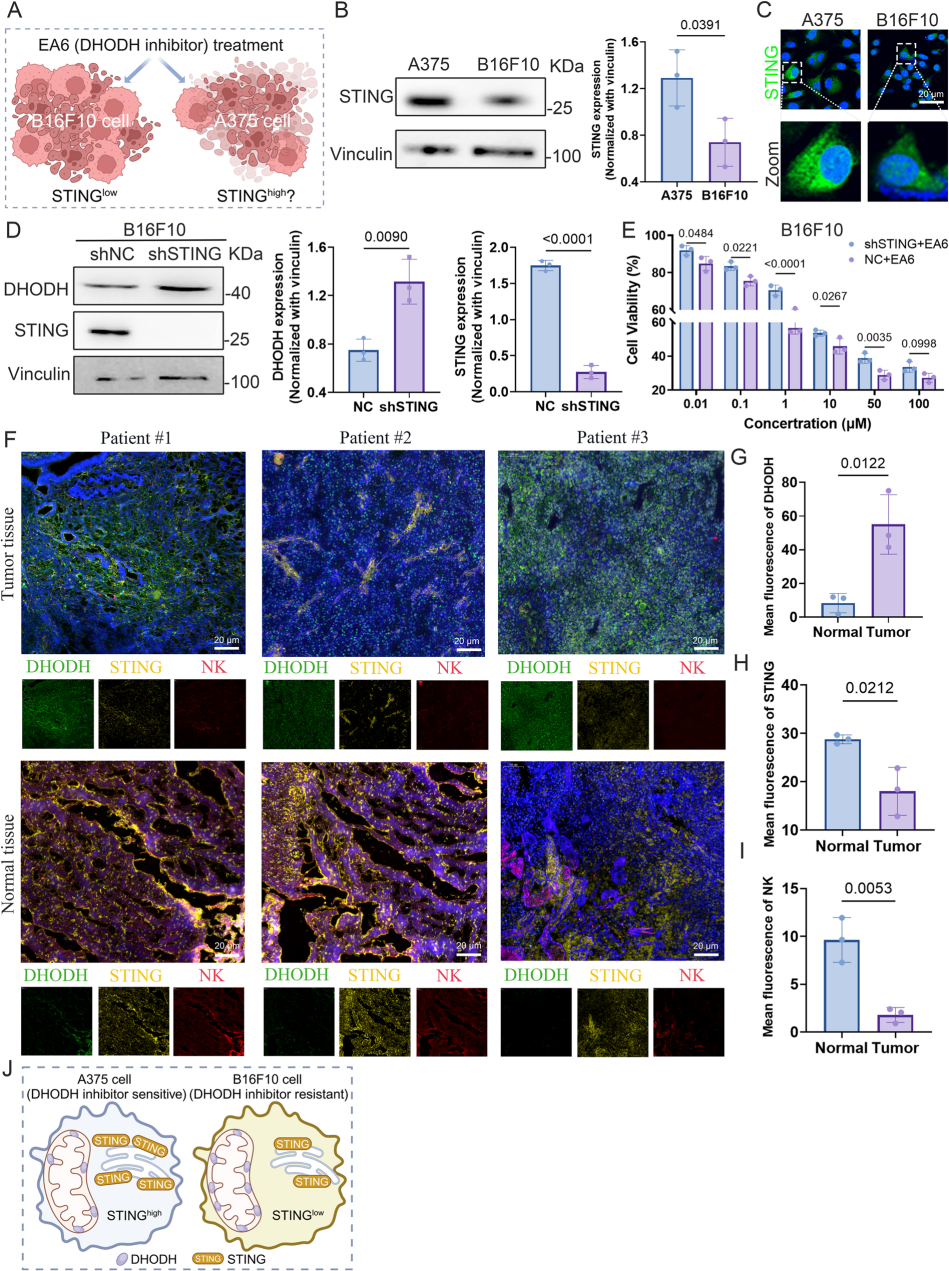
Low-expressed of STING mediates the therapeutic resistance to DHODH inhibitors. **A** Schematic illustration of scientific hypothesis. **B **Representative western blot images and quantitative analysis of STING expression in A375 and B16F10 cells. **C** Representative fluorescence images of STING expression in A375 and B16F10 cells. **D** Representative western blot images and quantitative analysis of the NC and shSTING groups in B16F10 cells. **E** Cell viability of the NC and shSTING groups in B16F10 cells treated with EA6. **F** Representative immunofluorescence staining images of tumor tissues and normal tissues from melanoma patients (Green fluorescence: DHODH, yellow fluorescence: STING, red fluorescence: NK cells). **G-I** Fluorescence quantitative analysis of **G**) DHODH, **H**) STING, and **I**) NK cells (*n* = 3). **J** Schematic diagram of DHODH inhibitor resistance mediated by low expression of STING

Sensitivity to DHODH inhibitors varies substantially among melanoma cell lines. In particular, B16F10 cells displayed marked resistance, while A375 cells were notably more responsive [25]. Our study identified that B16F10 cells possess markedly lower STING protein expression (STING^low^), prompting the hypothesis that diminished STING expression contributes to DHODH inhibitor resistance (Fig. 1A). Western blot analyses validated that STING levels were significantly reduced in B16F10 cells compared with A375 cells (Fig. 1B), a finding corroborated by confocal microscopy (Fig. 1C).

To assess whether STING directly modulates DHODH inhibitor sensitivity, we knocked down STING in B16F10 cells using short hairpin RNA (shSTING). STING silencing resulted in upregulated DHODH expression relative to the negative control (NC), suggesting a compensatory metabolic adaptation (Fig. 1D). Functionally, STING knockdown attenuated EA6-induced cytotoxicity, indicating that intact STING signaling contributes to the antitumor efficacy of DHODH inhibition (Fig. 1E).

We next examined STING and DHODH expression in human melanoma specimens and matched adjacent normal tissues (Fig. 1F). As anticipated, melanoma tissues exhibited elevated DHODH expression (Fig. 1G) and significantly diminished STING expression (Fig. 1H) relative to non-tumor counterparts. Prior work has demonstrated that EA6 facilitates NK cell infiltration via DHODH inhibition [12]. Consistent with this, patient samples showed a positive correlation between DHODH inhibition and NK cell recruitment (Fig. 1I).

Taken together, these findings indicate that low STING expression contributes to melanoma resistance to DHODH inhibitors. This further supports the hypothesis that restoring or enhancing the activity of the STING signaling pathway may re-sensitize drug-resistant tumors to DHODH-targeted therapy (Fig. 1J).

### In vitro antitumor activity of the prodrug H62

**Fig. 2:**
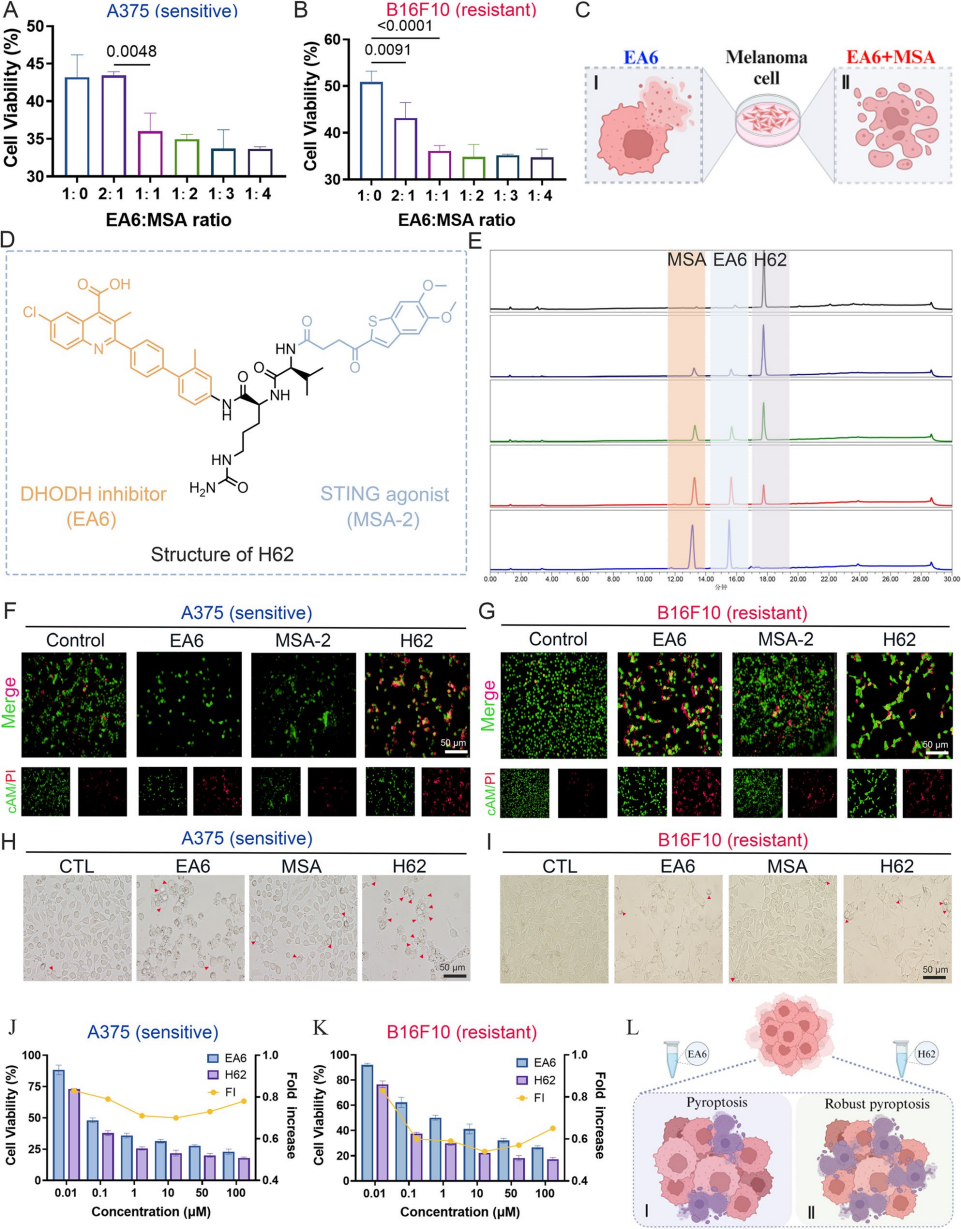
H62 effectively kills tumor cell. **A-B** The cell viability after administration of EA6 and MSA in different proportions in **A**) A375 and **B**) B16F10 cells (*n* = 3). **C** Scheme illustration of EA6/EA6 + MSA induced pyroptosis. **D** The structure of prodrug H62, where the blue part represents the STING agonist MSA, and the orange part represents DHODH inhibitor EA6. **E** The release ratio of prodrug H62 in a time dependent manner. **F-G** Live/dead cells staining images in **F**) A375 and **G**) B16F10 cells with DMSO, EA6, MSA, H62 for 24 h. **H-I** The representative images of cells morphology in A375 and B16F10 cells treated with indicated treatment. The red arrows denote that pyroptosis vesicles. **J-K** The cell viability of EA6 and H62 treated with **J**) A375 and **K**) B16F10 cells for 48 h (*n* = 3). **L **Schematic illustration of EA6 and H62 induced melanoma cells pyroptosis

To determine whether activation of the STING signaling pathway could enhance the antitumor efficacy of DHODH inhibitors, we first evaluated the combinatorial effects of EA6 (0.5 µM) and MSA, a well-characterized STING agonist, in A375 and B16F10 melanoma cells across varying molar ratios (Fig. 2A-B). Among the tested combinations, a 1:1 molar ratio of EA6 to MSA exhibited the most optimal cytotoxic effect and was therefore selected for subsequent studies (Fig. 2C).

To improve both therapeutic potency and tumor specificity, we next synthesized a CTSB-activatable prodrug, H62, by covalently linking EA6 and MSA via a CTSB-cleavable dipeptide linker (Fig. 2D). The route of H62 synthesis is shown in Scheme S1. CTSB is abundantly expressed in melanoma and other tumor types, making it an attractive enzymatic trigger for site-specific drug activation [26]. Structural validation of H62 was confirmed by ^1^H-NMR, ^13^C-NMR, and high-resolution mass spectrometry (HRMS), with full characterization presented in Fig. S1–S9. To characterize the metabolic profile of H62 in cells, we first analyzed its degradation kinetics in the presence of CTSB using high-performance liquid chromatography (HPLC). The results demonstrated that H62 undergoes efficient, time-dependent cleavage in the presence of CTSB, with near-complete release of EA6 and MSA within 6 h (Fig. 2E). This cleavage pattern is consistent with rapid enzymatic activation in the tumor microenvironment. To further validate this process under physiological conditions, we conducted additional experiments in B16F10 cells, which confirmed that endogenous CTSB similarly mediates the time-dependent cleavage of H62 (Fig. S1 0 A).

We next evaluated the antitumor potency of H62 in A375 and B16F10 cells. Live/dead staining revealed substantial induction of cell death following H62 treatment, with robust red propidium iodide (PI) fluorescence (Fig. 2F–G and Fig. S10B-C). Colony formation assays further demonstrated that H62 potently suppressed long-term cell proliferation compared with EA6 (Fig. S10D–E). Morphological assessment revealed numerous pyroptotic vesicles in H62-treated cells, suggestive of pyroptosis induction (Fig. 2H–I). Consistent with this, cell viability assays confirmed that H62 exhibited superior cytotoxic activity in both melanoma cell lines relative to EA6 alone (Fig. 2J–K).

To investigate the immunomodulatory capacity of H62, B16F10 cells were pre-treated with H62 for 24 h and then co-cultured with NK cells. The result indicating that the prodrug H62 can enhance the killing ability of NK cells against melanoma cells (Fig. S10F), indicating that H62 not only induces direct tumor cytotoxicity but also promotes immune-mediated clearance. Importantly, viability assays in NK92 cells confirmed that H62 was not intrinsically toxic to NK cells (Fig. S10G-H). Supporting the selectivity of the prodrug, western blot analysis revealed that NK92 cells expressed lower levels of DHODH compared to melanoma cells (Fig. S10I). To further evaluate the tumor-selective cytotoxicity of H62, we further assessed its effects on non-cancerous cells using a HUVEC model. Our results demonstrated that at the low concentration (0.5 µM) used in our in vitro experiments, H62 exhibited only minimal toxicity toward HUVEC cells (Fig. S10J). These results are consistent with existing evidence that tumor cells predominantly depend on *de novo* pyrimidine synthesis, thus rendering them uniquely vulnerable to DHODH inhibition.

Together, these results indicate that H62 robustly enhances the cytotoxic efficacy of DHODH inhibition in melanoma by integrating STING pathway activation, thereby sensitizing resistant tumor cells to pyroptosis while preserving NK cell viability (Fig. 2L).

### H62 induces pyroptosis and recruits NK cells to amplify tumor cell pyroptosis by inhibiting DHODH

Our previous studies demonstrated that the antitumor effect of EA6 is largely attributable to its ability to induce mitochondrial oxidative stress. To investigate whether prodrug H62 elicits similar mitochondrial dysfunction, we measured intracellular reactive oxygen species (ROS) levels using the CellRox probe. H62 treatment resulted in markedly elevated ROS accumulation in both A375 and B16F10 melanoma cells (Fig. S11A-B). Consistently, JC-1 staining revealed a notable shift from red to green fluorescence, indicating significant mitochondrial membrane depolarization following H62 exposure in both cell lines (Fig. S11C-D).

**Fig. 3:**
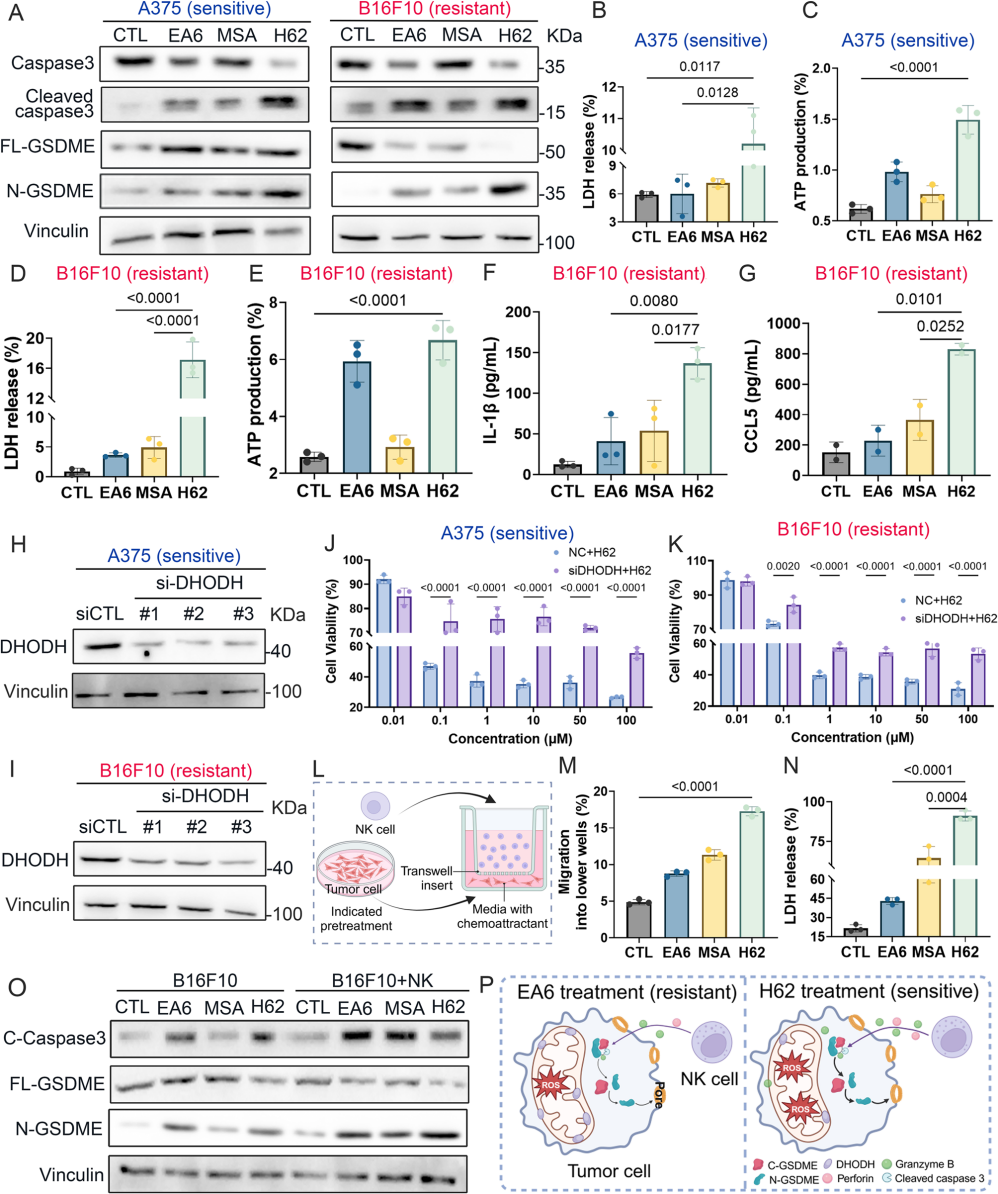
H62 induces tumor cell pyroptosis and recruits NK cell to amply pyroptosis by inhibiting DHODH. **A** The western bolt image of pyroptosis related protein in A375 and B16F10 cell lines, respectively. **B** The release of LDH after treated with DMSO, EA6, MSA and H62 in A375 cell (*n* = 3). **C** The production of ATP after incubation with various compounds in A375 cell (*n* = 3). **D** The release of LDH after treated with various compounds in B16F10 cell (*n* = 3). **E** The production of ATP after incubation with indicated treatment in B16F10 cell (*n* = 3). **F** The ELISA results of IL-1β secreted with indicated treatment in B16F10 cell (*n* = 3). **G** The ELISA results of CCL5 secreted by different compounds-treated B16F10 cell (*n* = 3). **H-I** Representative western blot images showing the expression of DHODH in **H**) A375 and **I**) B16F10 cells transfected with siRNA against DHODH. **J-K** Knockdown of DHODH expression the cytotoxicity of H62 in **J**) A375 and **K**) B16F10 cells. **L** Schematic illustration of transwell co-culture system (uper well: NK cell, lower well: tumor cell). **M** The migration ratio of NK cells into lower wells when co-culture B16F10 and NK cells for 6 h (*n* = 3). **N** The release of LDH after co-culturing B16F10 and NK cells (*n* = 3). **O** The western blot images of pyroptosis related protein after co-culturing B16F10 and NK cells for 8 h. **P** Schematic illustration of H62 induce pyroptosis and recruit NK cell to amply tumor cell pyroptosis

Excessive ROS generation, caspase 3 was activated to cleave GSDME, and N-GSDME formed pores on the cell membrane, inducing cell pyroptosis while producing large amounts of cytokines [27, 28]. Therefore, we further checked the occurrence of cell pyroptosis with different treatments. Western blot analysis showed that after H62 treatment, the expression of cleaved caspase 3 and N-GSDME, which are pyroptosis-related proteins, increased in melanoma cell lines (Fig. 3A). Additionally, another characteristic of pyroptosis is the release of cellular contents [29]. The results showed that lactate dehydrogenase (LDH) release increased in A375 and B16F10 cells following H62 treatment (Fig. 3B, D). ATP, a type of damage-associated molecule pattern (DAMP), which can be released from pyroptotic cells [30], was significantly increased in the H62 treatment group (Fig. 3C, E**)**. Furthermore, ELISA results confirmed that H62 treatment induced the production of more of the inflammatory factor IL-1β associated with pyroptosis (Fig. 3F). CCL5 is a chemokine that has been reported to induce NK cell infiltration into tumors [31]. ELISA results showed a significant increase in CCL5 in cell culture supernatant after H62 treatment (Fig. 3G).

To validate that the antitumor activity of H62 is mediated by inhibiting DHODH, we knocked down DHODH expression in A375 and B16F10 cells to confirm the crucial role of DHODH in H62-induced tumor cell death. Then, the knockdown effect of the DHODH protein was verified via western blot (Fig. 3H-I). As expected, cell viability was dramatically reversed after DHODH was silenced in both melanoma cell lines (Fig. 3J-K). Besides, the mitochondrial damage induced by H62 was partially restored after silencing DHODH, including a decrease in ROS level and an increase in mitochondrial member potential (MMP) (Fig. S11E-H). Similarly, supplementation with exogenous uridine—a salvage-pathway precursor that bypasses *de novo* pyrimidine synthesis—rescued cell viability in H62-treated cells [32] (Fig. S11I-J), reinforcing the role of pyrimidine depletion in mediating H62’s cytotoxicity. To further investigate the mechanism of H62-induced cell death, we treated the cells with the ferroptosis inhibitor Ferrostatin-1 (Fer-1). The results showed that Fer-1 could effectively reverse the killing effect of H62, thus confirming that H62 can also induce ferroptosis in tumor cells (Fig. S11K-L).

Previous findings from our group indicated that EA6-induced pyroptosis promotes NK cell infiltration, creating a feed-forward loop that amplifies tumor cell death. To determine whether H62 elicits a similar effect, we employed a transwell migration assay, with NK cells seeded in the upper chamber and H62-pretreated B16F10 cells in the lower chamber (Fig. 3L). NK cell migration was significantly increased toward H62-conditioned melanoma cells (Fig. 3M). Co-culture of NK cells with H62-pretreated tumor cells further resulted in elevated LDH release from NK cells (Fig. 3N), suggesting enhanced NK cell activation. Additionally, western blotting revealed increased expression of pyroptosis-associated proteins in co-cultures, compared to H62-treated B16F10 cells cultured alone (Fig. 3O).

Collectively, these findings demonstrate that prodrug H62 overcomes melanoma resistance to DHODH inhibition by simultaneously inducing pyroptosis and recruiting NK cells, thereby amplifying pyroptotic cell death through integrated STING activation and targeted DHODH inhibition (Fig. 3P).

#### H62 activates the STING pathway and enhances NK cell activation

**Fig. 4:**
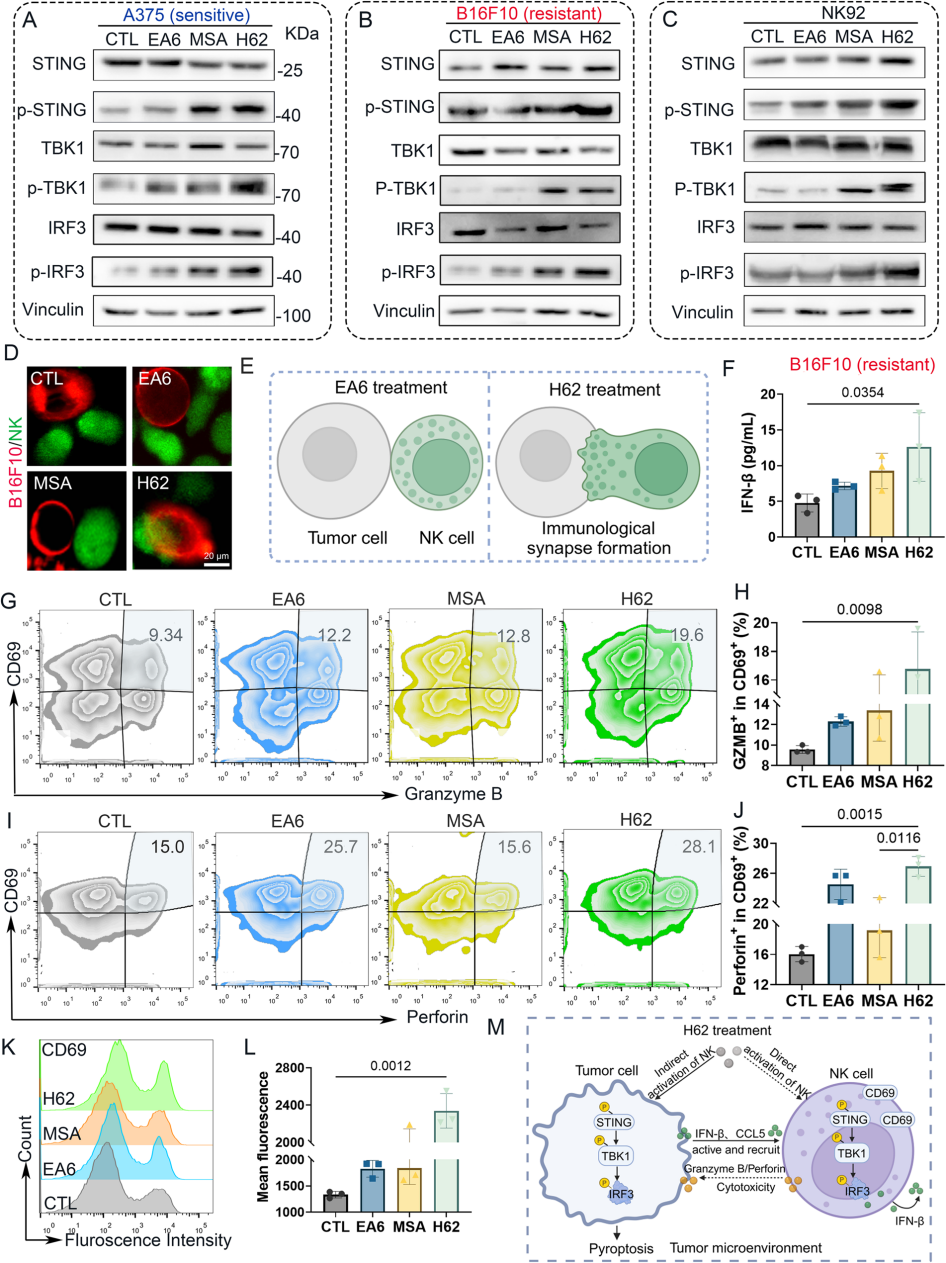
H62 activates STING pathway and promotes NK cells activation in vitro. **A-C** The western blot images of STING signaling pathway related protein in **A**) A375, **B**) B16F10 and **C**) NK92 cells. **D** The representative images of IS. **E** Schematic illustration of IS treated with different compounds. **F** The ELISA results of IFN-β secreted by different compounds-treated B16F10 cell (*n* = 3). **G** Granzyme B flow cytometry analysis of co-culture NK cell and different pre-treatment B16F10 cells (*n* = 3). **H** The quantitative analysis results of Granzyme B. **I** Perforin flow cytometry analysis of co-culture NK cells and different pre-treatment B16F10 cells (*n* = 3). **J** The quantitative analysis results of perforin. **K** CD69 flow cytometry analysis of co-culture NK92 cells and different pre-treatment B16F10 cells (*n* = 3). **L** The flow cytometry mean fluorescence of CD69. **M** Schematic illustration of H62 activates STING pathway and enhances NK cells activation

To validate that the antitumor efficacy of prodrug H62 is mediated through activation of the STING signaling cascade via the release of MSA, we performed western blot analysis to assess key STING pathway components, including STING, TANK-binding kinase 1 (TBK1), and interferon regulatory factor 3 (IRF3). H62 treatment led to a marked increase in phosphorylated STING (p-STING), TBK1 (p-TBK1), and IRF3 (p-IRF3) levels in A375 cells (Fig. 4A and Fig. S12A). Similar upregulation of these phosphorylated signaling intermediates was also observed in both B16F10 melanoma cells and NK92 cells following H62 exposure (Fig. 4B-C, Fig. S12B-C), confirming STING pathway activation in both tumor and immune effector cells.

The immunological synapse (IS) — a specialized junction formed between NK cells and their target cells—is essential for directed cytolytic granule release and effective tumor cell killing [33, 34]. To investigate whether H62 enhances IS formation, NK cells were co-cultured with B16F10 cells pretreated with H62 for 6 h, followed by CLSM analysis. H62 treatment significantly increased the formation of IS compared to EA6-treated controls (Fig. 4D-E), suggesting enhanced NK–tumor cell engagement.

We next quantified IFN-β production as a downstream functional readout of STING activation. H62 treatment significantly elevated IFN-β secretion (Fig. 4F), consistent with robust activation of the cGAS–STING–IFN-I axis. In tumor cells, STING activation induces IFN-I and chemokine secretion, which together promote NK cell recruitment and functional maturation [35]. In parallel, STING signaling in NK cells themselves enhances the release of cytolytic mediators, including perforin and granzyme B, thereby potentiating their tumor-killing capacity [36, 37].

Notably, granzyme B also facilitates the cleavage of GSDME into its active N-terminal fragment (N-GSDME), amplifying pyroptosis in tumor cells [38]. To further explore the impact of H62 on NK cell effector function, we assessed granzyme B expression following co-culture of NK cells with B16F10 cells pretreated with EA6 or H62. Granzyme B levels were significantly higher in the H62 group than in the EA6 group (Fig. 4G-H). Similarly, perforin production was markedly increased upon H62 treatment (Fig. 4I-J). Expression of CD69, a canonical marker of NK cell activation [39], was also notably upregulated in response to H62 (Fig. 4K-L).

To directly test the role of STING in mediating H62’s antitumor effects, we silenced STING expression in B16F10 cells and treated them with H62. STING knockdown markedly attenuated the cytotoxicity induced by H62 (Fig. S12D-E), confirming that the STING signaling pathway is indispensable for H62-mediated tumor cell death. Similarly, knockdown of STING expression in A375 cells also significantly reversed the tumor-killing effect of H62 (Fig. S12F).

In summary, these findings establish that prodrug H62 activates the STING pathway in melanoma and NK cells, thereby enhancing NK cell cytotoxicity and promoting the secretion of key effector molecules, including perforin and granzyme B (Fig. 4M).

### H62 exhibits potent antitumor activity in vivo

**Fig. 5:**
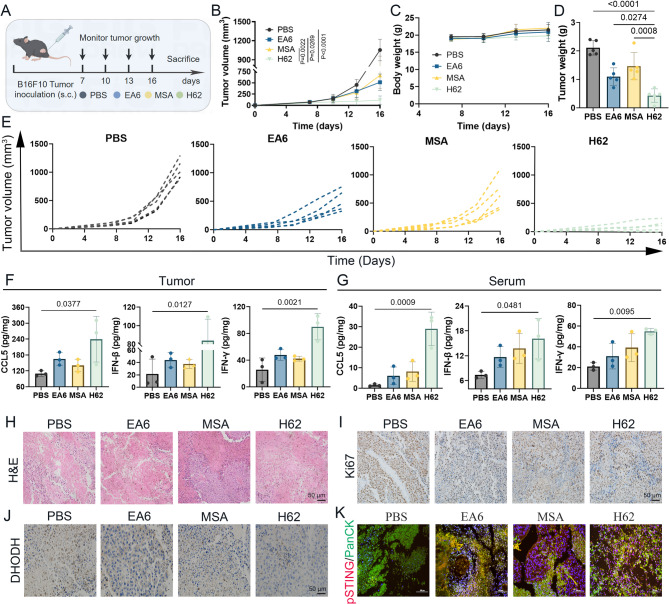
Anti-tumor effect of various compounds in vivo. **A** Schematic representation of H62 anti-tumor efficacy in B16F10 subcutaneous tumor-bearing model. **B** Tumor volume of different treatments group during experiment (*n* = 5). **C** Body weight record of each group mice during experiment (*n* = 5). **D** Tumor weight of different administration at the end of the experiment (*n* = 5). **E** Individual tumor volume of each group mice during experiment (*n* = 5). **F** The ELISA of results various indicators of tumor tissue after various administration treatments (*n* = 3). **G** The ELISA of results various indicators of serum following various administration treatments (*n* = 3). **H-K** Representative images of H) H&E, I) Ki-67, J) DHODH and K) p-STING staining of tumor tissues

To evaluate the in vivo antitumor efficacy of prodrug H62, we established a subcutaneous B16F10 melanoma model. Once tumors reached a volume of approximately 50 mm³, mice were randomly allocated into four treatment groups: PBS, EA6 (30 mg/kg), MSA (30 mg/kg), and H62 (30 mg/kg). All agents were administered intraperitoneally according to the schedule outlined in Fig. 5A. Among these, H62 elicited the most pronounced tumor growth inhibition compared to monotherapy groups (Fig. 5B), with detailed tumor growth curves presented in Fig. 5E. Importantly, no significant differences in body weight were observed across groups during treatment (Fig. 5C), indicating no apparent adverse effects.

At the experimental endpoint, tumors were excised, imaged, and weighed. Mice treated with H62 exhibited substantially reduced tumor burdens compared to those receiving EA6 or MSA alone (Fig. 5D). Cytokine profiling revealed significantly elevated levels of CCL5, IFN-β, and IFN-γ in both tumor tissues and serum of H62-treated mice (Fig. 5F-G), consistent with an enhanced systemic and local immune response.

The in vivo pharmacokinetic profile of a drug plays a crucial role in determining its therapeutic efficacy and safety. Therefore, we conducted a systematic evaluation of the pharmacokinetic characteristics of H62. The results showed that the elimination half-life (t_1/2_) of H62 was approximately 10.043 h, indicating more superior pharmacokinetic properties compared with EA6 (Fig. S13A). Histological examination using hematoxylin and eosin (H&E) staining revealed extensive nuclear fragmentation and cytolysis in tumors from the H62 group, far exceeding the pathological changes observed in EA6 and MSA treated cohorts (Fig. 5H). Moreover, Ki-67 immunostaining showed a marked reduction in proliferative activity following H62 treatment (Fig. 5I). Immunohistochemical staining for DHODH, along with immunofluorescence detection of p-STING, demonstrated a dual effect: DHODH expression was significantly suppressed, while STING pathway activation was strongly enhanced by H62 administration (Fig. 5J-K). Consistent with the induction of pyroptosis observed in vitro, western blot analysis confirmed increased expression of GSDME in tumor tissues from H62-treated mice (Fig. S13B).

Survival analysis further supported the therapeutic benefit of H62, as mice in this group exhibited significantly prolonged survival relative to controls (Fig. S13C). To evaluate systemic safety, we performed H&E staining of major organs and conducted routine hematological assessments. No overt histopathological abnormalities or hematologic toxicity were observed across treatment groups (Fig. S13D–P), indicating that H62 does not induce measurable systemic toxicity under the conditions tested.

Together, these data provide compelling in vivo evidence that the prodrug H62 robustly overcomes melanoma resistance to DHODH inhibitors by concurrently targeting mitochondrial pyrimidine metabolism and activating the STING signaling axis, achieving potent antitumor efficacy with no apparent adverse effects.

### H62 induces potent antitumor immune response

**Fig. 6:**
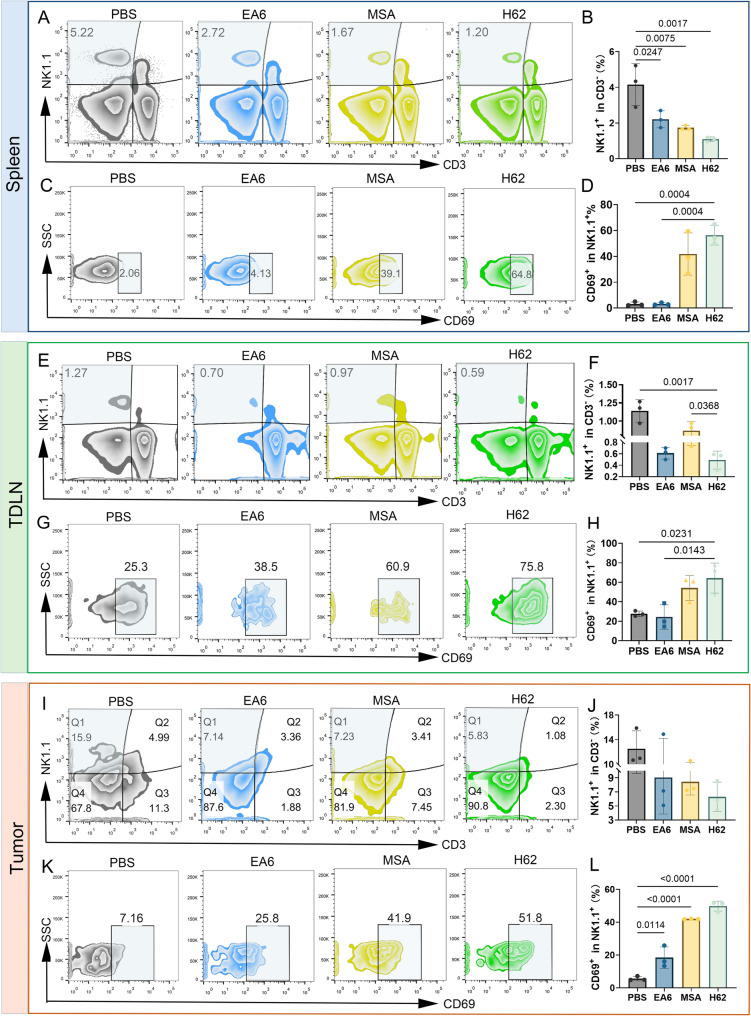
H62 recruits and activates NK cell in vivo. **A-B** Flow cytometric analysis of NK (CD3^−^NK1.1^+^) and **C-D** CD69^+^ cells in spleens of mice in various groups. **E-F** Flow cytometric analysis of NK (CD3^−^NK1.1^+^) and **G-H** CD69^+^ cells in TDLNs of mice in various groups. **I-J** Flow cytometric analysis of NK (CD3^−^NK1.1^+^) and **K-L **CD69^+^ cells in tumors of mice in various groups

To further elucidate the immunomodulatory effects of prodrug H62 in vivo, we examined its impact on two critical immune compartments: the spleen and tumor-draining lymph nodes (TDLNs). Flow cytometric analysis revealed a marked alteration in NK cell dynamics following H62 treatment. In the spleen, the proportion of CD3⁻NK1.1⁺ NK cells declined sharply to 1.08%, representing an approximately fourfold reduction relative to the PBS control group (4.15%) (Fig. 6A–B). This decrease is consistent with prior reports and likely reflects the redistribution of activated NK cells from lymphoid reservoirs to peripheral tumor sites [40–42]. Concomitantly, the proportion of activated NK cells, as indicated by CD69 expression, increased significantly in the spleens of H62-treated mice, reaching 56.3% (Fig. 6C–D), suggesting robust systemic NK cell activation.

Similar trends were observed in TDLNs. While the overall frequency of CD3⁻NK1.1⁺ NK cells was reduced following H62 treatment (Fig. 6E–F), the proportion of CD69⁺ activated NK cells was markedly elevated (Fig. 6G–H), indicating enhanced NK cell priming within regional immune niches. Although the total number of tumor-infiltrating NK cells (CD3⁻NK1.1⁺) remained relatively unchanged across groups (Fig. 6I–J), the activation status of these intratumoral NK cells was significantly enhanced by H62. The percentage of CD69⁺ NK cells rose to 49.8% in the H62 group, higher than the PBS (44.2%), EA6 (31.4%), and MSA (7.9%) groups, respectively (Fig. 6K–L). These data suggest that H62 not only facilitates NK cell mobilization but also potently enhances their effector activation state within the tumor microenvironment.

The above results suggest that prodrug H62 robustly promotes the recruitment and functional activation of NK cells in multiple immunological compartments—including the spleen, TDLNs, and tumor—thereby significantly amplifying innate immune responses against melanoma.

### H62’s antitumor effect depends on DHODH Inhibition and STING-mediated NK cell activation

**Fig. 7:**
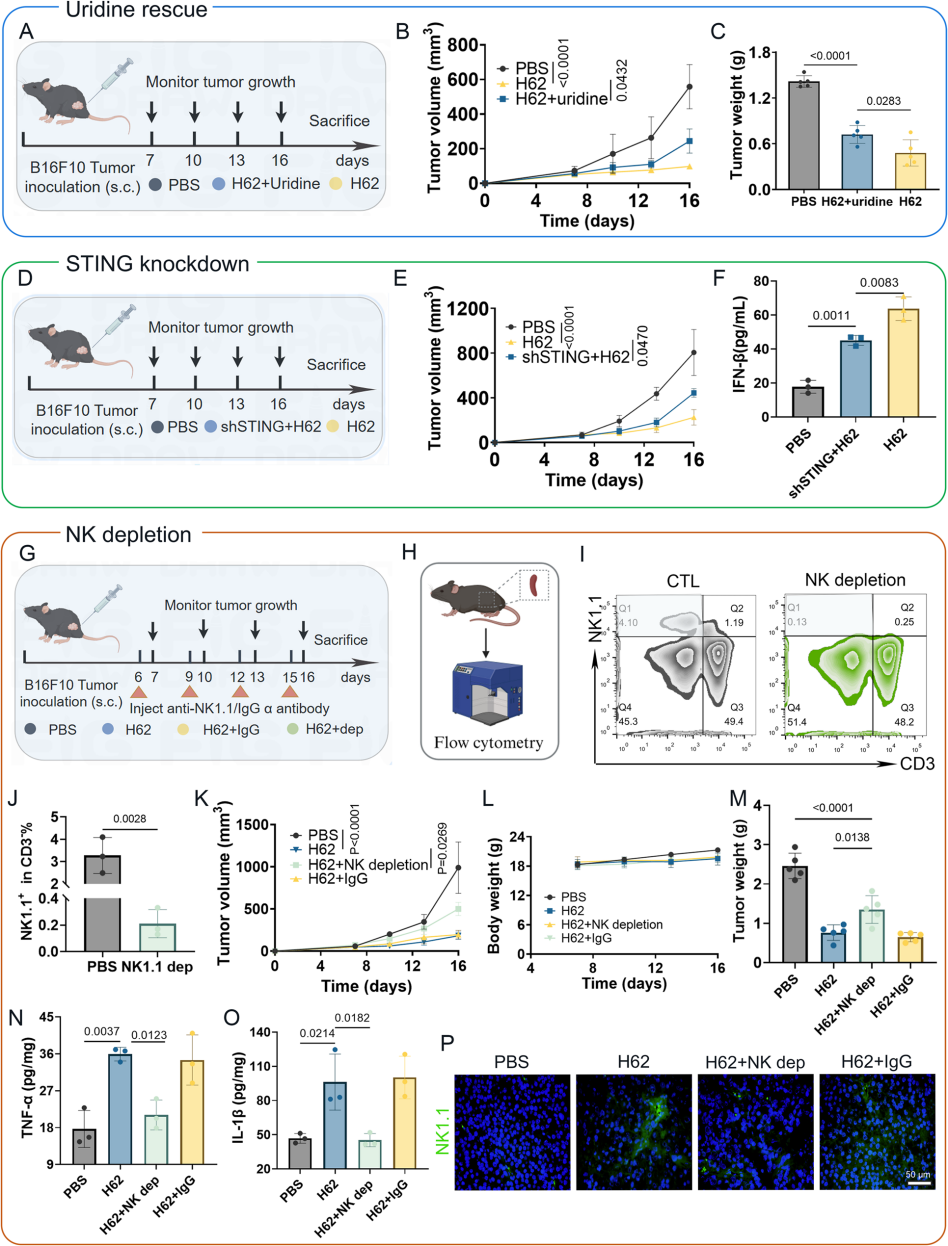
The antitumor mechanism of H62 in vivo. **A** Schematic representation of a uridine rescue experiment. **B** Tumor growth curve of different treatment group (*n* = 5). **C** Tumor weight of various group mice (*n* = 5). **D** Schematic representation of the STING knockdown experiment. **E** Tumor growth curves in different experimental groups (*n* = 5). **F** Serum IFN-β levels in various experimental groups (*n* = 3). **G** Experiment scheme for prodrug H62 therapy with NK cells depletion (**H**) The flowchart for the NK cell depletion verification experiment. **I** Flow cytometery verification of in vivo clearance effect of anti-NK cell antibody. **J** Statistical analysis of NK cell depletion efficiency (*n* = 3). **K** Tumor growth curves in various treatment groups (*n* = 5). **L** Monitoring of mice body weight in NK cell depletion experiment (*n* = 5). **M** Tumor weight in different experimental groups (*n* = 5). **N** Tumor tissues TNF-α content in different groups (*n* = 3). **O** Serum IL-1β level in different treatment groups (*n* = 3). **P** The representative image of NK1.1 staining of tumor tissue

To clarify the mechanistic basis underlying the antitumor effects of prodrug H62 in vivo, we first assessed whether its activity depends on DHODH inhibition. A B16F10 melanoma xenograft model was established, and mice were treated with PBS, H62, or H62 in combination with exogenous uridine (H62 + Uridine), which bypasses the blockade of *de novo* pyrimidine synthesis (Fig. 7A). Tumor volume analysis revealed that uridine supplementation significantly abrogated the tumor-suppressive effect of H62, indicating that DHODH inhibition is a critical component of H62’s efficacy (Fig. 7B and Fig. S14A–B). Consistently, final tumor weights in the H62-treated group were substantially lower than those in the H62 + Uridine group (Fig. 7C). Body weights remained stable across all treatment groups, indicating good tolerability (Fig. S14C).

Next, to further validate the contribution of STING signaling to the antitumor mechanism of H62, we employed a genetic knockdown model. B16F10 cells stably transduced with short hairpin RNA targeting STING (shSTING) or a negative control vector were implanted into C57BL/6 mice, followed by intraperitoneal administration of H62 (Fig. 7D). STING knockdown significantly impaired the antitumor efficacy of H62 compared to the wild-type control, with tumors exhibiting accelerated growth and reduced responsiveness (Fig. 7E and Fig. S14D). Again, no notable differences in body weight were observed across groups (Figure S14E). Additionally, serum IFN-β levels—used as a surrogate marker for STING pathway activation—were markedly reduced in the shSTING + H62 group relative to the H62 group, confirming effective suppression of STING signaling (Fig. 7F and Fig. S14F).

Finally, to directly evaluate the role of NK cells in mediating H62-induced tumor control, we performed in vivo NK cell depletion using a neutralizing anti-NK1.1 antibody (Fig. 7G). Flow cytometry confirmed efficient depletion of NK cells in treated animals (Fig. 7H–J). As expected, NK cell depletion reversed the antitumor effect of H62, leading to significantly increased tumor burden relative to H62 treatment alone (Fig. 7K and Fig. S14G–H), without affecting systemic toxicity or body weight (Fig. 7L). Consistent with tumor growth data, the H62 group showed the lowest tumor weights, which were significantly increased following NK cell depletion (Fig. 7M). Cytokine analysis further revealed that TNF-α and IL-1β—key effectors of NK-mediated tumor cell killing and pyroptosis—were substantially reduced in the absence of NK cells [43] (Fig. 7N–O). Immunofluorescence staining of NK1.1 confirmed enhanced NK cell infiltration in tumors treated with H62, which was notably diminished upon NK cell depletion (Fig. 7P).

These results demonstrate that the antitumor efficacy of prodrug H62 is driven by a dual mechanism involving DHODH inhibition and STING pathway activation. This dual targeting strategy triggers tumor cell pyroptosis and promotes the recruitment and activation of NK cells, establishing an immunometabolic feedback loop that effectively suppresses melanoma progression in vivo.

### H62 enhances neoadjuvant and immune checkpoint blockade efficacy

Given that H62 robustly promotes NK cell infiltration and extends survival in melanoma-bearing mice, we hypothesized that its administration in a neoadjuvant setting could further improve therapeutic outcomes by reducing postoperative tumor recurrence. Neoadjuvant therapy, which includes systemic interventions such as chemotherapy, targeted therapy, and immunotherapy administered prior to surgical resection, aims to shrink primary tumors and eradicate micrometastatic disease to improve long-term survival [44–46].

**Fig. 8:**
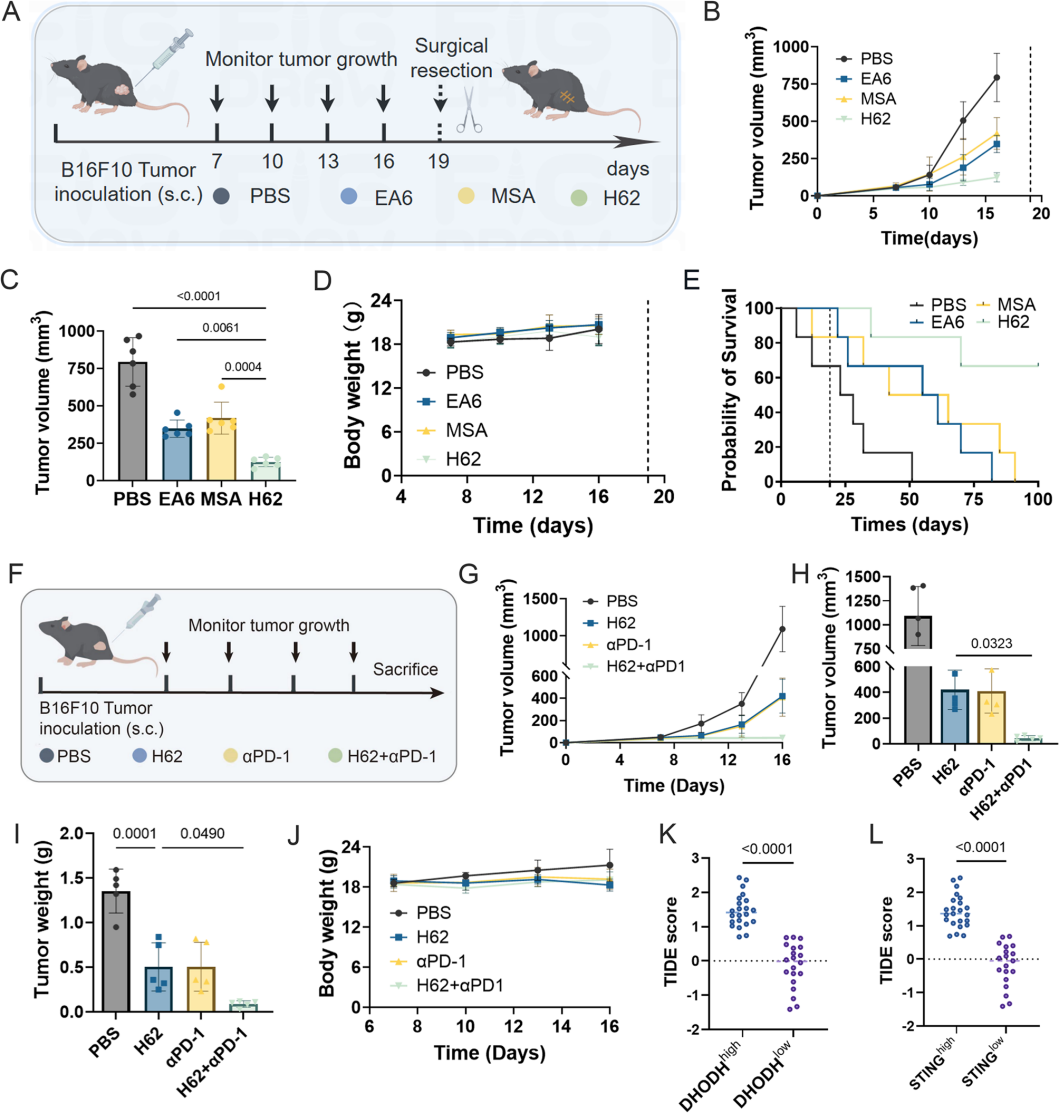
H62 enhances neoadjuvant and immune checkpoint blockade efficacy. **A** Schematic representation of H62 neoadjuvant therapy efficacy in B16F10 subcutaneous tumor-bearing model. **B** Tumor growth curves of various treatment groups before surgical resection of tumors (*n* = 6). **C** Tumor volume of different treatment groups on day 16 (*n* = 6). **D** The change of body weight of various groups before surgical resection of tumors (*n* = 6). **E** The survival period of different group mice after surgical resection of tumors (*n* = 6). **F** Schematic representation of synergistic therapy in B16F10 subcutaneous tumor-bearing model. **G** Tumor growth curves of various treatment groups (*n* = 5). **H** Tumor volume of different treatment groups on day 16 (*n* = 5). **I** Tumor weight in different experimental groups (*n* = 5). **J** Monitoring of mice body weight (*n* = 5). **K** DHODH expression level and TIDE score in melanoma patients (DHODH^high^, *n* = 22; DHODH^low^, *n* = 20). **L **STING expression level and TIDE score in melanoma patients (STING^high^, *n* = 23; STING^low^, *n* = 19)

To evaluate this possibility, we employed a B16F10 melanoma murine model and administered PBS, EA6, MSA, or H62 in four doses prior to tumor excision (Fig. 8A). Tumor burden was monitored during the preoperative phase. H62-treated mice exhibited significantly slower tumor growth compared with the other groups, indicating superior tumor control prior to surgery (Fig. 8B–C). Importantly, no significant differences in body weight were observed between groups throughout the treatment period, suggesting good tolerability (Fig. 8D).

Following surgical resection, we monitored recurrence and overall survival. Mice pre-treated with H62 showed markedly reduced tumor relapse and extended survival compared with those treated with EA6, MSA, or PBS (Fig. 8E). These results indicate that the immunometabolic remodeling induced by H62 not only enhances primary tumor response but also reduces residual disease and metastatic potential post-resection.

To evaluate the potential of H62 in enhancing immune checkpoint blockade (ICB) therapy, we conducted a combination treatment of H62 with αPD-1 in B16F10 tumor-bearing mice (Fig. 8F). The results demonstrated that H62 significantly enhanced the therapeutic efficacy of αPD-1 monotherapy (Fig. 8G-J). Of note, to further explore the potential clinical relevance of the DHODH/STING axis in melanoma immunotherapy response, we performed a bioinformatic analysis using the publicly available TCGA dataset. The results demonstrated that suppression of DHODH expression significantly lowered the tumor immune dysfunction and exclusion (TIDE) score (Fig. 8K), implying a weakened response to ICB therapy. Conversely, activation of the STING pathway with STING agonists markedly increased the TIDE score (Fig. 8L), suggesting enhanced sensitivity to immunotherapy. These findings indicate that combined DHODH inhibition and STING activation may synergistically improve the efficacy of immune checkpoint inhibitors, which is consistent with the experimental observations from our animal models and strengthens the clinical relevance of our study.

Taken together, our findings demonstrate that H62 confers substantial benefit in a neoadjuvant therapeutic setting by enhancing preoperative tumor control, limiting recurrence, and improving overall survival. Moreover, H62 can effectively enhance the efficacy of the immune checkpoint inhibitor αPD-1 in the melanoma model.

## Discussion

Metabolic reprogramming is a hallmark of malignant transformation, contributing not only to sustained tumor proliferation and therapeutic resistance, but also to immune evasion [47]. Among the various metabolic pathways co-opted by cancer cells, *de novo* pyrimidine biosynthesis—principally regulated by the mitochondrial enzyme DHODH—has emerged as a tractable metabolic vulnerability [48]. DHODH facilitates nucleotide production to support rapid cell division and links mitochondrial metabolism to immune suppression within the TME [49]. Although multiple DHODH inhibitors have advanced into preclinical and clinical testing, their efficacy in solid tumors remains limited, primarily due to intrinsic or acquired resistance mechanisms [50].

Building on our prior findings that pharmacological DHODH inhibition induces mitochondrial oxidative stress and pyroptotic cell death in melanoma—alongside recruitment of NK cells—we previously developed EA6, a next-generation DHODH inhibitor with enhanced potency. EA6 exhibited robust cytotoxic activity and NK cell recruitment in sensitive melanoma models [12]. However, heterogeneous responses across melanoma subtypes, particularly poor efficacy in B16F10 cells, highlighted a critical resistance phenotype that constrains the broader applicability of DHODH-targeted strategies [25, 51].

In this study, we identified low basal expression of STING as a key molecular correlate of resistance to DHODH inhibition in melanoma. This finding prompted the hypothesis that restoring STING signaling could overcome resistance by enhancing NK cell activity within the TME. To test this, we rationally designed the tumor-selective prodrug H62 by covalently linking EA6 with the STING agonist MSA via a CTSB-cleavable peptide linker. This design enables synchronized and tumor-specific release of both therapeutic agents within the protease-enriched TME.

H62 demonstrated significantly enhanced antitumor efficacy over either monotherapy arm in both in vitro and in vivo settings, most notably in DHODH inhibitor–refractory B16F10 models. Mechanistically, DHODH inhibition by H62 disrupted mitochondrial homeostasis, leading to increased ROS, caspase-3 activation, and cleavage of GSDME, which together initiated pyroptotic tumor cell death. This was accompanied by release of DAMPs, including ATP and IL-1β, as well as increased CCL5 secretion—factors that collectively supported a pro-inflammatory microenvironment conducive to NK cell recruitment.

Beyond recruitment, H62-mediated STING activation substantially augmented NK cell functional responses. This included increased IFN-I production, upregulation of activation markers such as CD69, enhanced immunological synapse formation with target cells, and increased secretion of granzyme B and perforin. Notably, granzyme B further contributed to pyroptosis via cleavage of GSDME, creating a feedforward loop that amplified tumor cell death. These results underscore the capacity of H62 to engage both tumor-intrinsic and immune-mediated mechanisms to achieve durable antitumor responses.

In melanoma in vivo models, H62 markedly suppressed tumor growth and prolonged survival. Functional dissection revealed that each component—DHODH inhibition, STING activation, and NK cell activity—was essential for full therapeutic efficacy. Disruption of any arm (via uridine supplementation, STING knockdown, or NK cell depletion) substantially attenuated treatment benefit. Significantly, H62 was shown to provide substantial survival benefits and reduce tumor recurrence in a neoadjuvant therapy. Furthermore, combining H62 with αPD-1 produced synergistic anti-tumor effects, providing theoretical and experimental support for its potential for translation into clinical practice.

Despite these advances, several questions remain. First, while H62 robustly activated NK cells, the metabolic-immune crosstalk that governs NK cell differentiation, activation thresholds, and functional exhaustion in the context of altered pyrimidine metabolism remains poorly defined. Elucidating these dynamics may uncover additional levers to fine-tune immunometabolic interventions. Second, although prior data suggest that EA6 exerts its effects predominantly through NK cell engagement, it is not yet clear whether H62’s immunomodulatory activity extends to other immune cell populations, including dendritic cells, macrophages, and T lymphocytes. Single-cell transcriptomic and spatial proteomic approaches will be instrumental in delineating the broader immune remodeling landscape induced by dual DHODH/STING targeting.

## Conclusions

In this study, we identified STING expression as a key molecular determinant of melanoma sensitivity to DHODH inhibition. To overcome resistance and enhance antitumor immunity, we rationally engineered the tumor-activated prodrug H62, which combines a potent DHODH inhibitor (EA6) with a validated STING agonist (MSA) via a protease-cleavable linker. H62 effectively triggered tumor pyroptosis and NK cell recruitment via DHODH inhibition, while concurrently activating NK cells through STING signaling. In both standard and neoadjuvant treatment settings, H62 significantly suppressed tumor growth, reduced recurrence, and extended survival in preclinical melanoma models. These findings establish H62 as a promising dual-function immunometabolic agent with the potential to overcome resistance to DHODH inhibition and improve therapeutic outcomes in immune-refractory malignancies.

## Supplementary Information

Supplementary Material 1.

Supplementary Material 2.

## Data Availability

Data is provided within the manuscript or supplementary information files.

## Abbreviations

DHODH: Dihydroorotate dehydrogenase
NK: Natural killer
IFN-I: Type I interferons
STING: Stimulator of interferon genes
MDSC: Myeloid-derived suppressor cells
TME: Tumor microenvironment
CTSB: Cathepsin B
HRMS: High-resolution mass spectrometry
HPLC: High-performance liquid chromatography
IHC: Immunohistochemistry
PI: Propidium iodide
ROS: Reactive oxygen species
LDH: Lactate dehydrogenase
DAMP: Damage-associated molecule pattern
MMP: Mitochondrial member potential
TBK1: TANK-binding kinase 1
IRF3: Interferon regulatory factor 3
P-STING: Phosphorylated STING
CLSM: Confocal laser scanning microscopy
IS: Immunological synapse
H&E: Hematoxylin and eosin
TDLNs: Tumor-draining lymph nodes
ICB: Immune checkpoint blockade
TIDE: Tumor immune dysfunction and exclusion
