# Supplementary material for: An immunometabolic prodrug strategy overcomes DHODH inhibitor resistance in refractory melanoma
Source: J Exp Clin Cancer Res. 2025 Nov 14;44:306. doi: 10.1186/s13046-025-03566-6 (PMC12619403; doi:10.1186/s13046-025-03566-6)
Supplement: Supplementary file 1 — Supplementary Material 1. [file 13046_2025_3566_MOESM1_ESM.pdf]

Supporting Information

Title:

An Immunometabolic Prodrug Strategy Overcomes DHODH Inhibitor Resistance in Refractory Melanoma

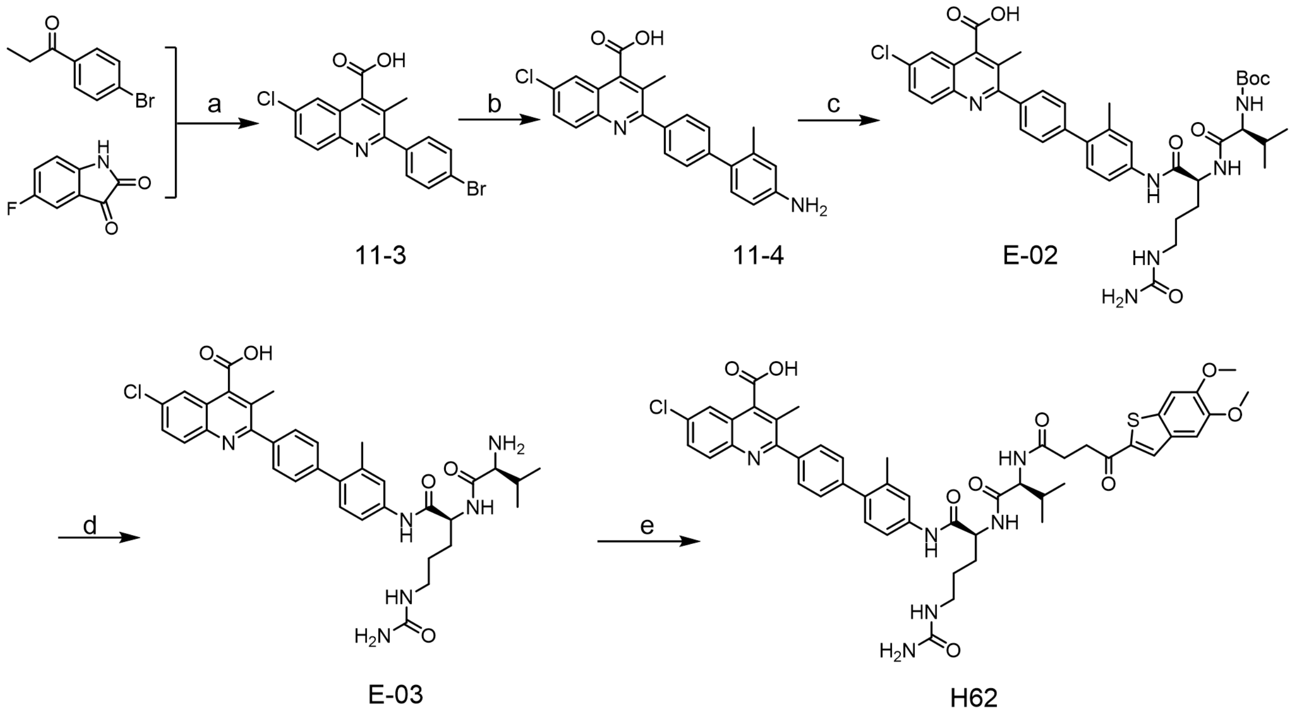

Scheme S1 Synthesis route of H62**.** Reaction and conditions: (**a**) KOH, EtOH, H_2O, EtOAc; (**b**) $Pd(PPh_3)_4$, Na_2CO_3, dioxane, H_2O, EtOAc; (**c**) TFA, HATU, DIPEA, DCM; (**d**) MeOH, H_2O, HCl, dioxane; (**e**) DCM, DIPEA.

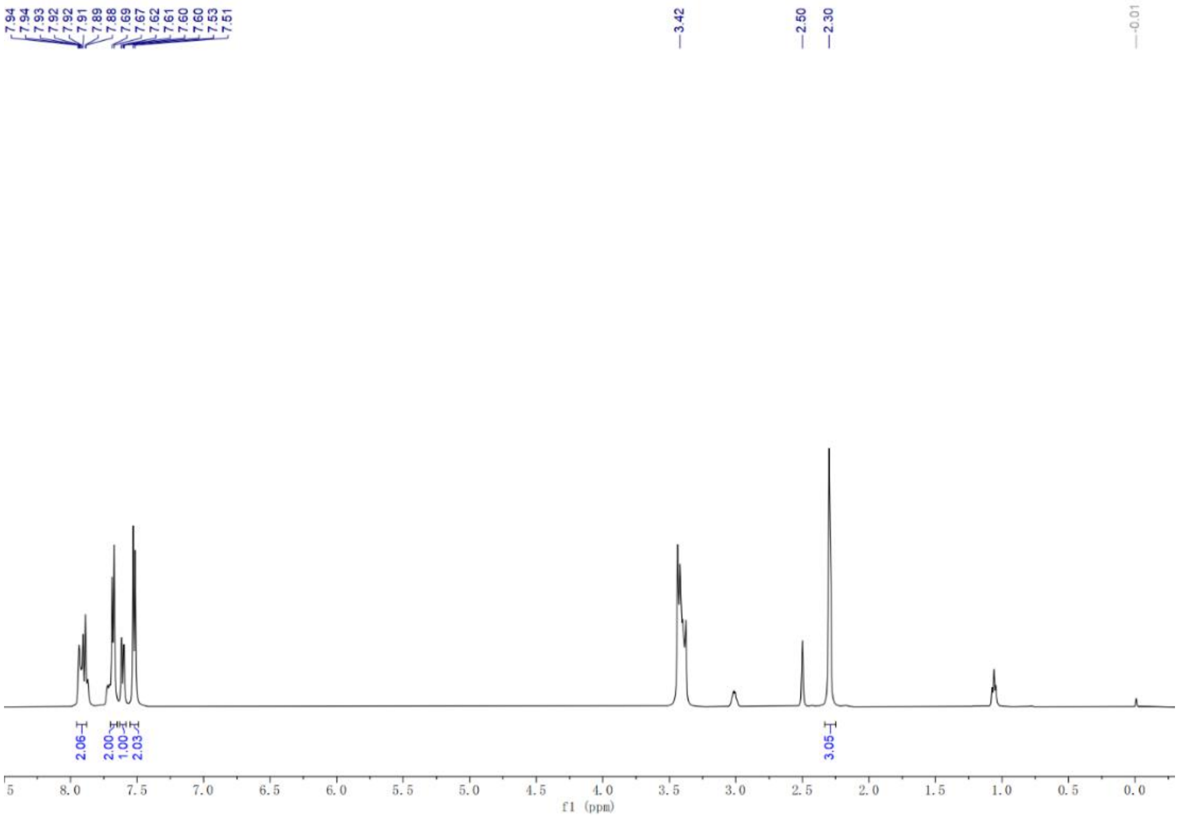

Fig. S1 ^{1}H-NMR spectrum of intermediate **11-3**

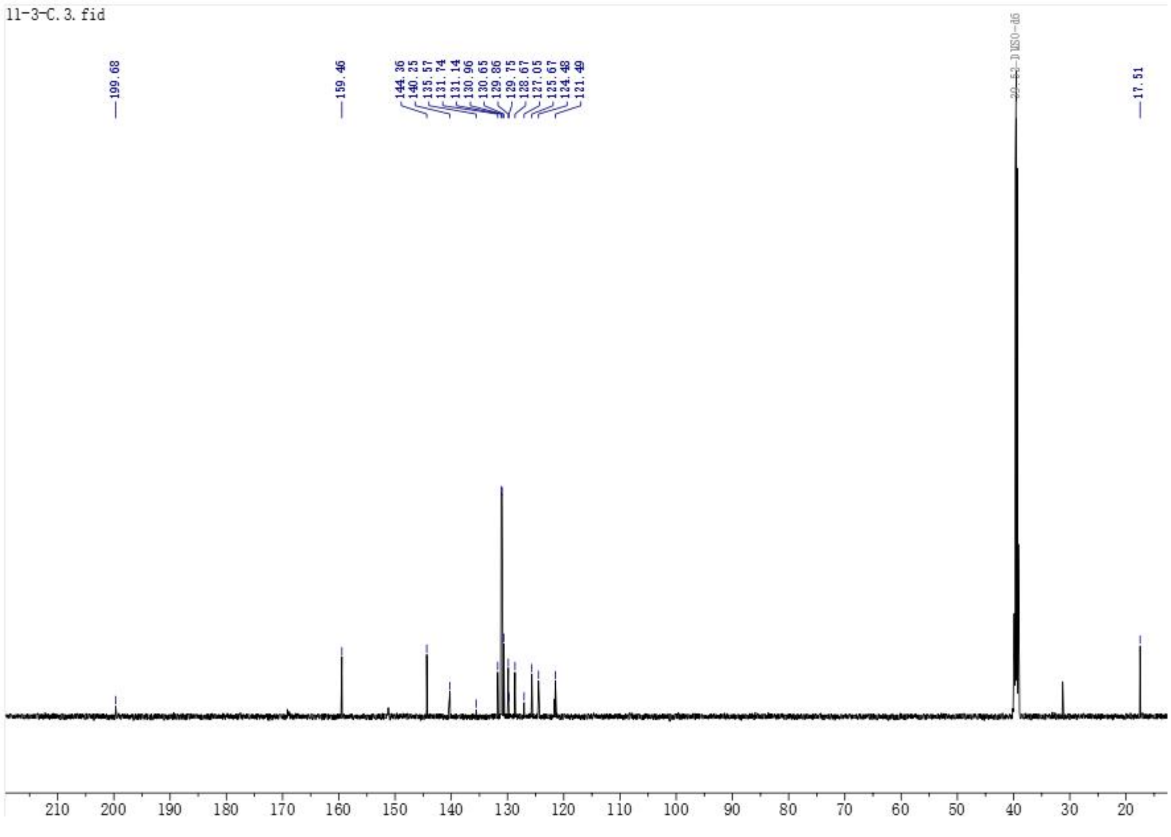

Fig. S2 ^{13}C-NMR spectrum of intermediate **11-3**

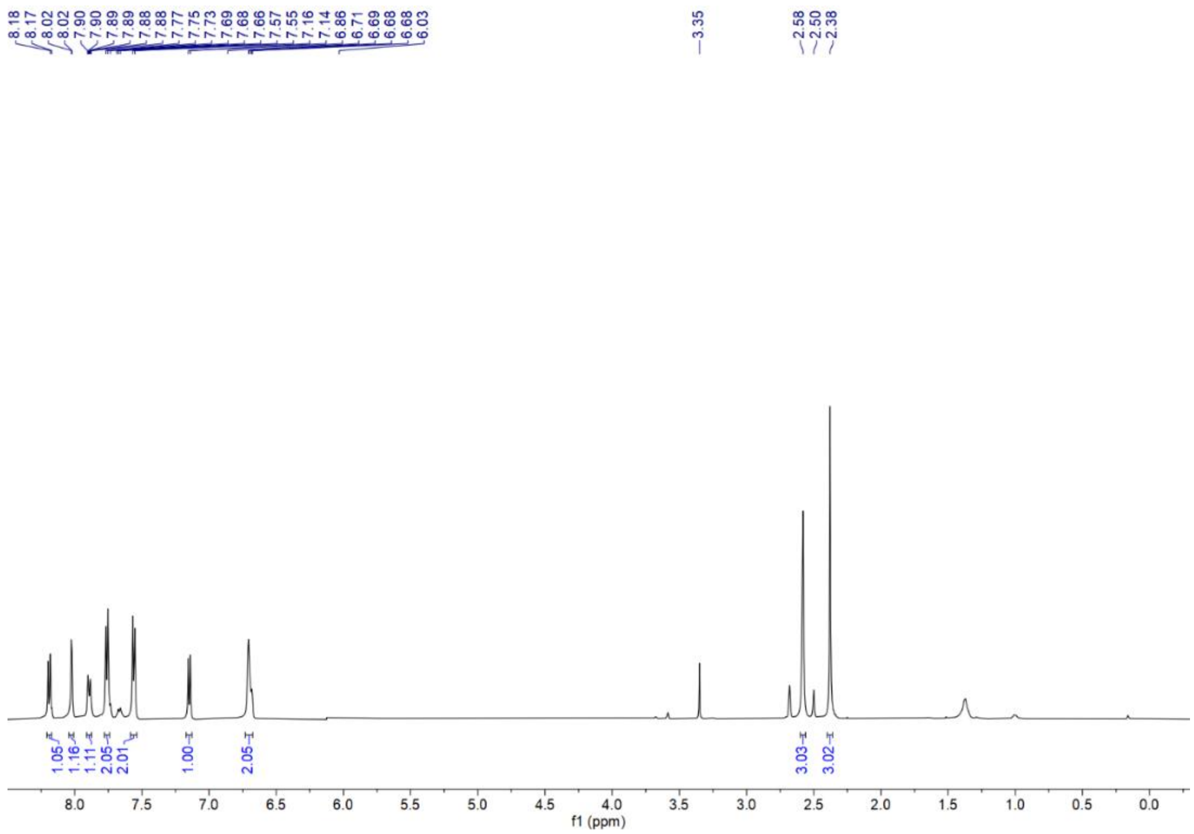

Fig. S3 ^{1}H-NMR spectrum of intermediate **11-4**

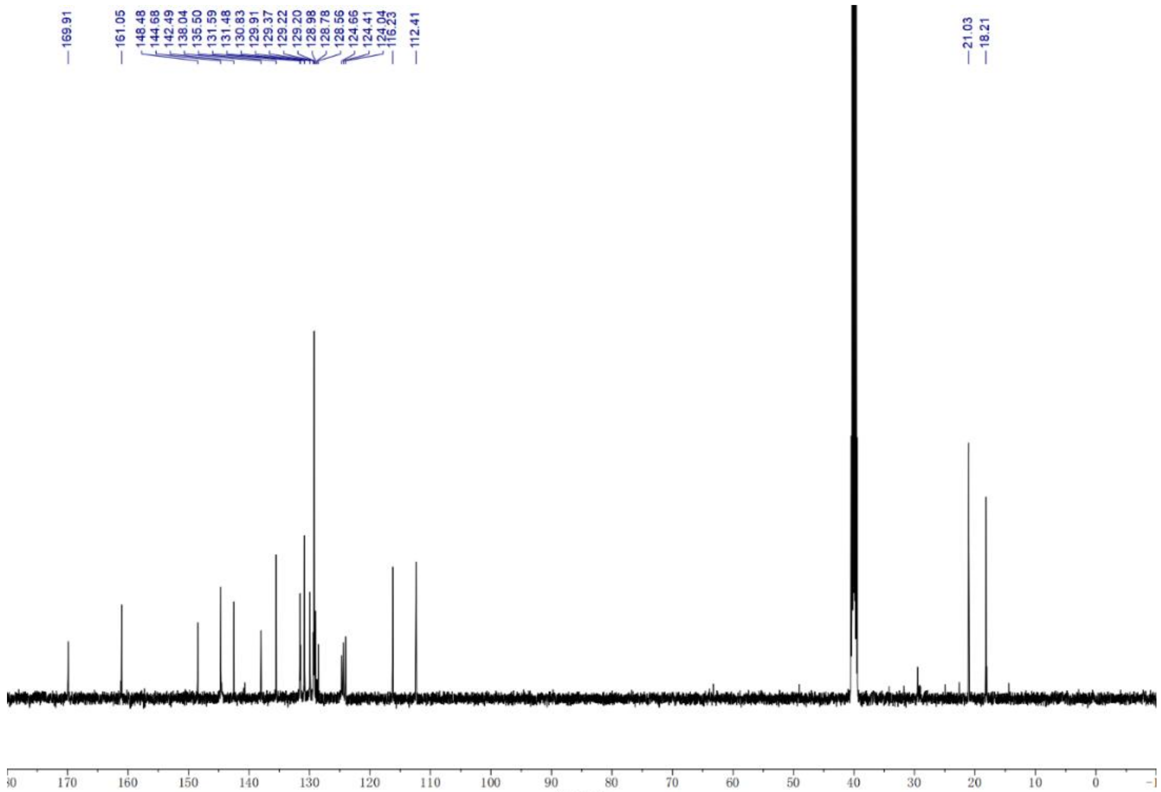

Fig. S4 ^{13}C-NMR spectrum of intermediate **11-4**

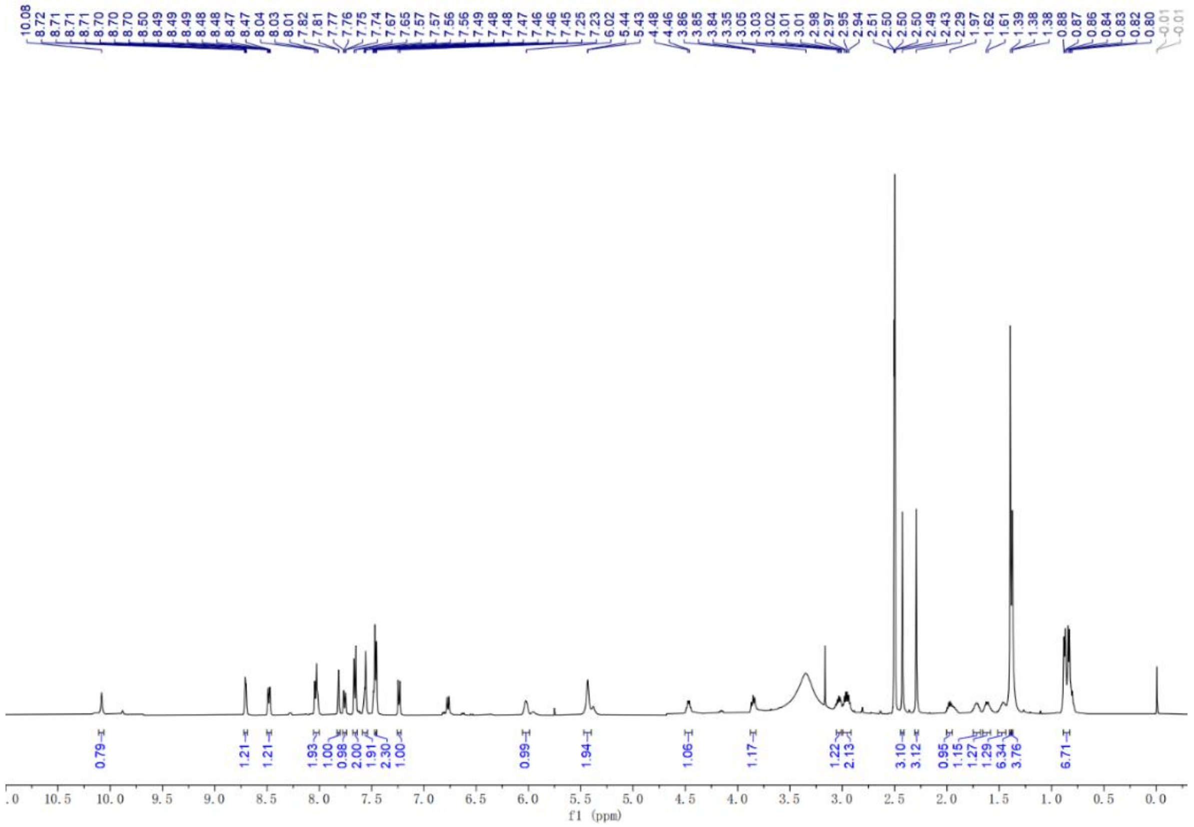

Fig. S5 ^{1}H-NMR spectrum for intermediate **E-02**

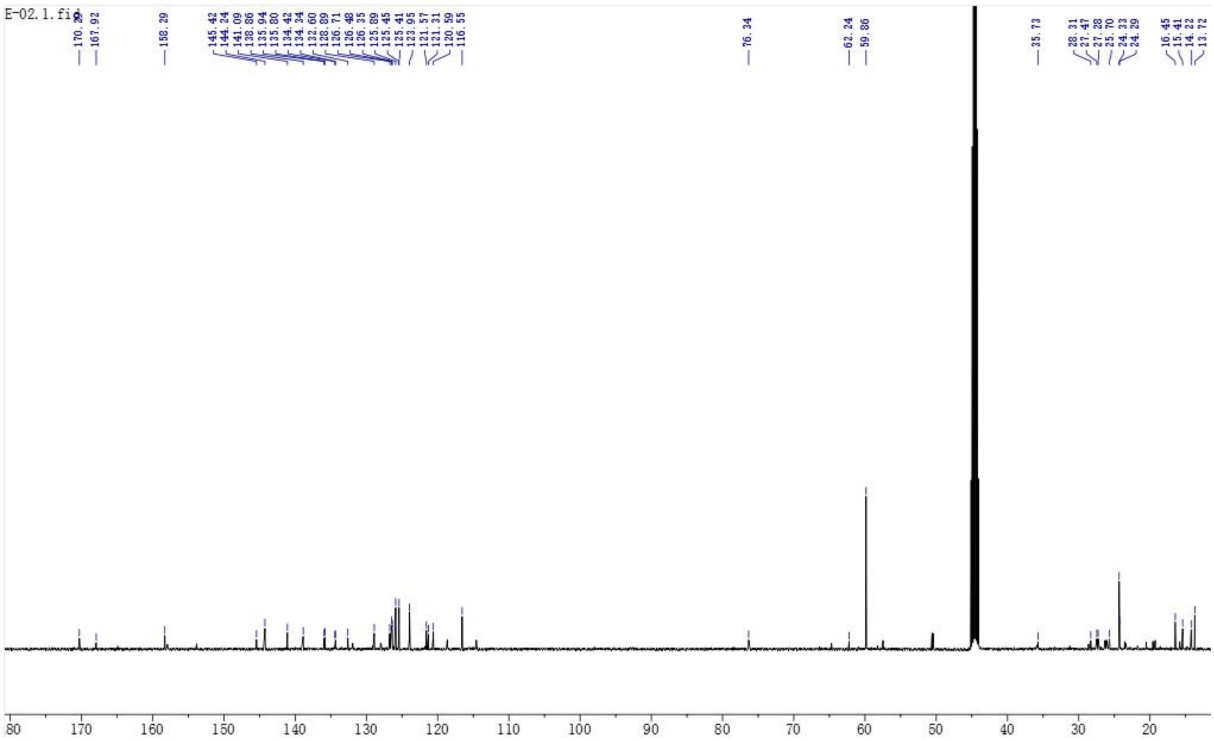

Fig. S6 ^{13}C-NMR spectrum of intermediate **E-02**

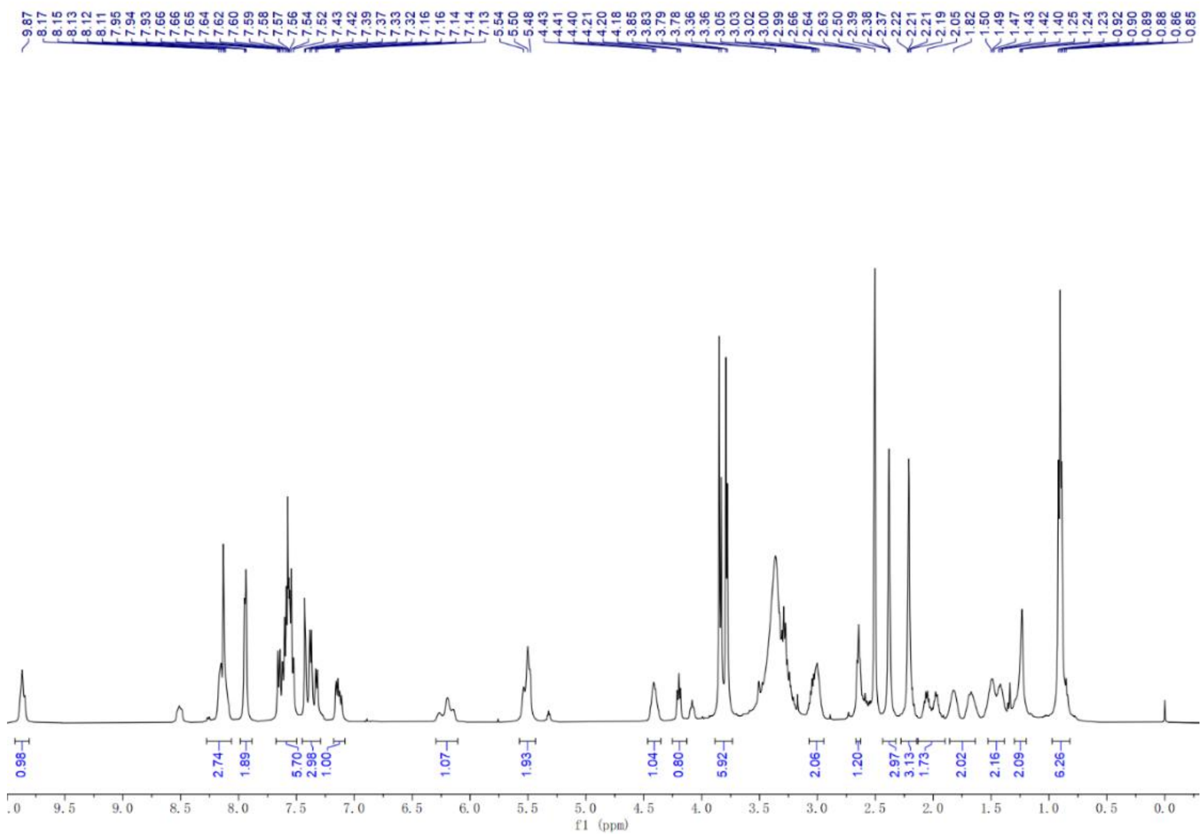

Figure. S7. ^{1}H-NMR spectrum of **H62**

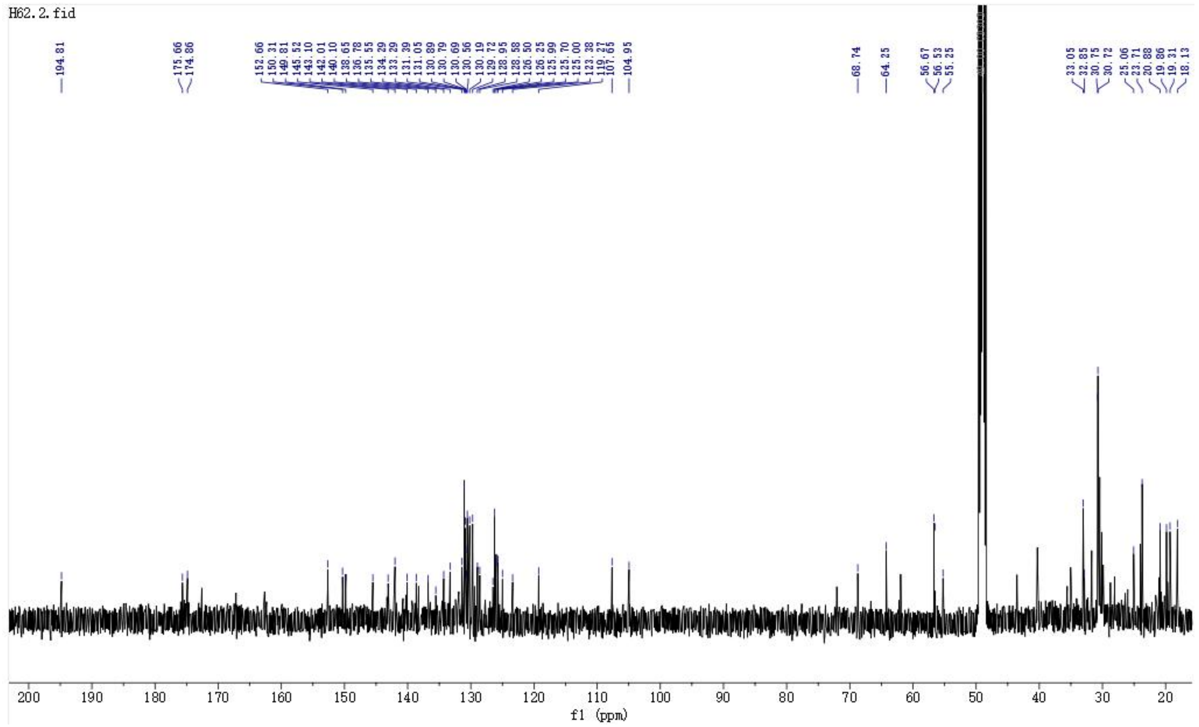

Fig. S8 ^{13}C-NMR spectrum of **H62**

Monoisotopic Mass, Even Electron Ions
26 formula(e) evaluated with 3 results within limits (all results (up to 1000) for each mass)
Elements Used:
C: 9-50 H: 13-53 N: 4-6 O: 3-10 S: 0-1 Cl: 1-1
20241227--GF29 16 (0.162)
1: TOF MS ES+

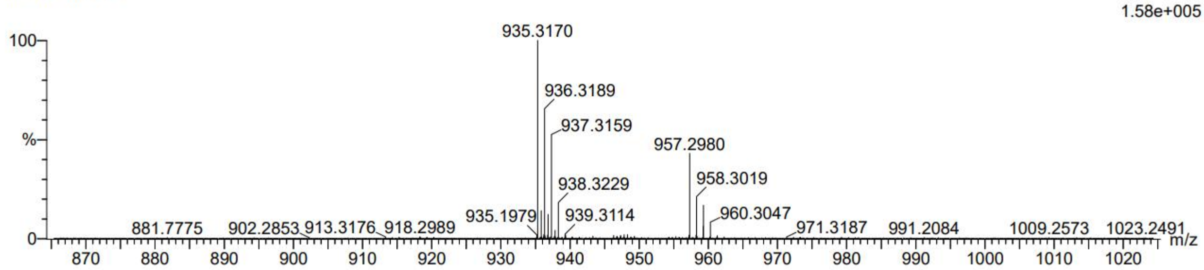

Minimum:				-1.5				
Maximum:		5.0	50.0	100.0				

Mass	Calc. Mass	mDa	PPM	DBE	i-FIT	Norm	Conf(%)	Formula
935.3170	935.3205	-3.5	-3.7	26.5	925.3	1.023	35.95	C49 H52 N6 O9 S Cl
	935.3093	7.7	8.2	26.5	925.5	1.204	29.98	C50 H52 N4 O10 S Cl
	935.2841	32.9	35.2	27.5	925.4	1.077	34.07	C48 H48 N6 O10 S Cl

Fig. S9 HRMS spectrum of **H62**

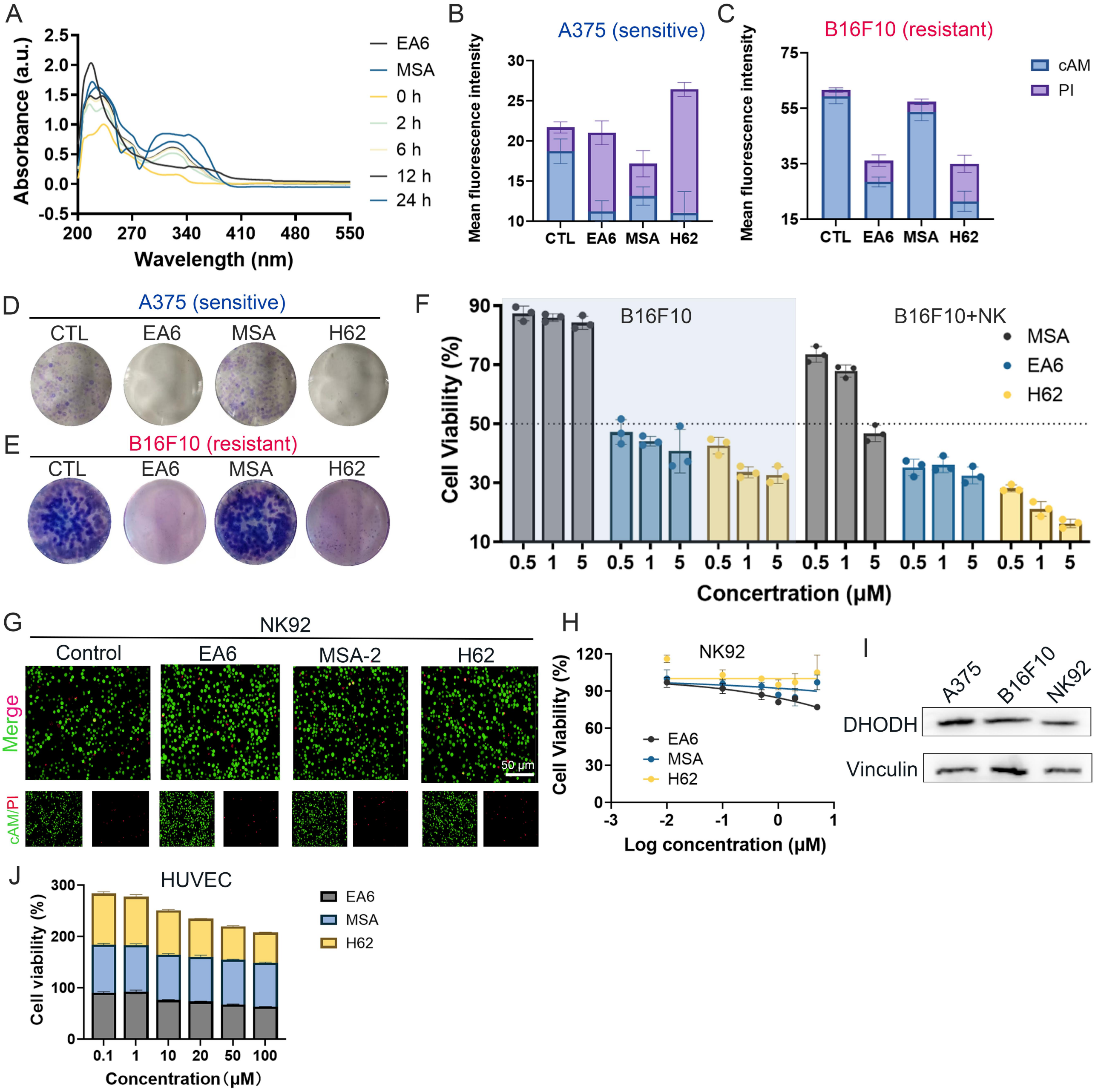

Fig. S10 The H62 mediates specific killing tumor cells *in vitro*. (**A**) UV-Vis absorption spectra of compounds EA6, MSA, and H62 after different treatments of B16F10 cells. (**B-C**) Quantification analysis of cAM/PI in B) A375 and C) B16F10 cell lines with different treatment (n = 3). (**D-E**) Cell colony formation assay in A375 and B16F10 cells, respectively. (**F**) The cell viability of B16F10 cells after 24 h of pretreatment with different compounds, followed by 6 h of co-culture with or without NK cells (n = 3). (**G**) Live/dead cells staining with different treatments in NK92 cells. Scale bar = 50 μm. (**H**) The cell viability at various concentrations for 24 h in NK92 cell (n = 3). (**I**) The western blot images of DHODH expression in A375, B16F10 and NK92 cells. (**J**) The cell viability at various concentrations in HUVEC cell (n = 3).

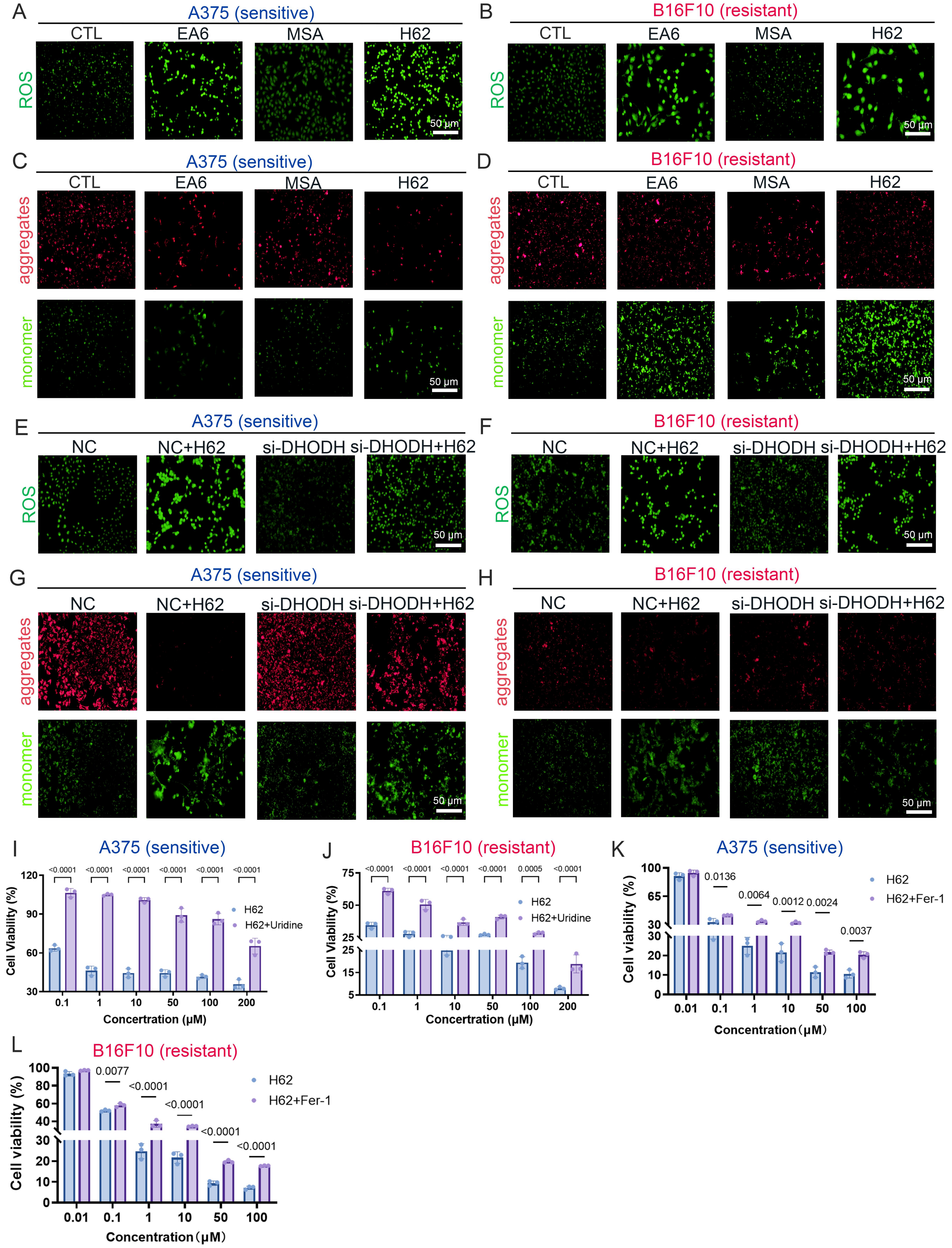

Fig. S11 H62 induces DHODH mediated tumor cell pyroptosis and NK cell recruitment. (**A-B**) Representative images of intracellular ROS generation in A375 and B16F10 cells stained with

CellRox probe after various treatments. Scale = 50 µm. (**C-D**) Representative images of mitochondrial membrane potential in A375 and B16F10 cells stained with JC-1 probe after various treatments for 24 h. Scale = 50 µm. (**E-F**) The production of ROS stained with CellRox probe after various treatment in A375 and B16F10 cells. Scale = 50 µm. (**G-H**) Representative images of mitochondrial membrane potential in A375 and B16F10 cells stained with JC-1 probe after various treatments for 24 h. Scale = 50 µm. (**I-J**) The cell viability of A375 and B16F10 cells after H62-treatment and add or without add uridine (100 µM) at different concentration for 48 h (n = 3). (**K-L**) Cell viability in K) A375 and L) B16F10 cells treated with H62 or H62+Fer-1 (n = 3).

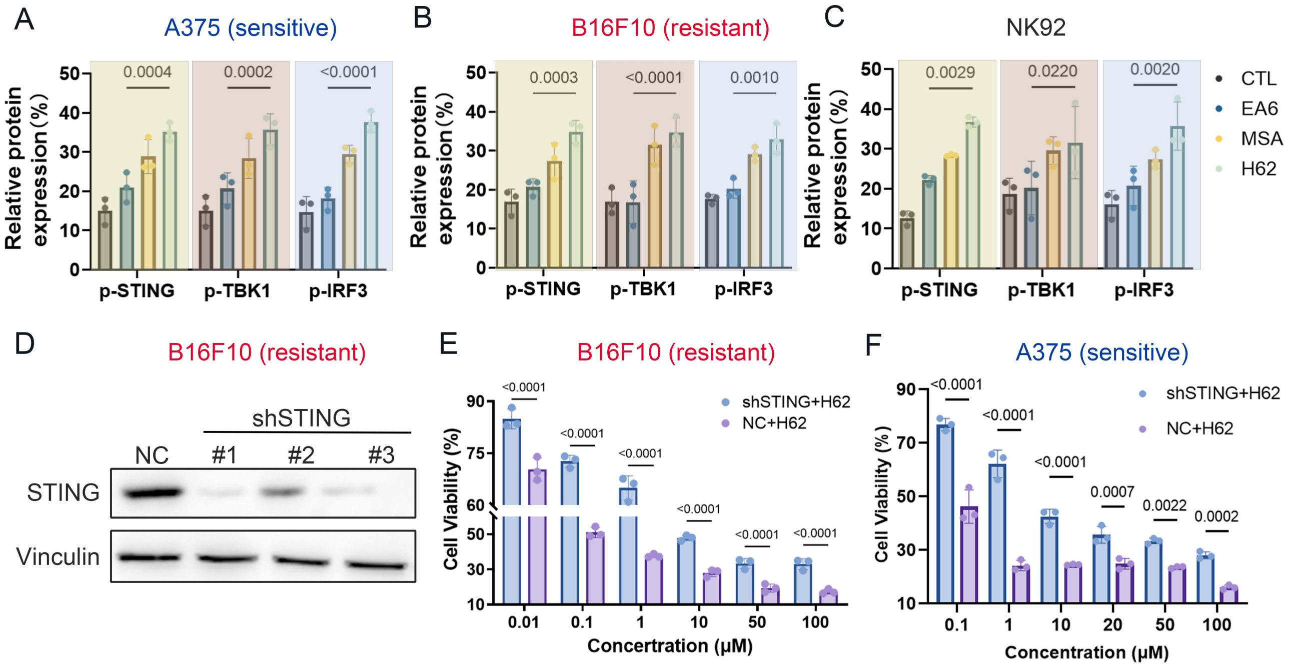

Fig. S12 H62 activates the STING signaling pathway to enhance responsiveness to DHODH inhibitors. (**A-C**) Quantitative analysis of p-STING, p-TBK1 and p-IRF3 expression in A) A375 B) B16F10 and C) NK92cells (n = 3). (**D**) The representative western blot image of knocking down STING in B16F10 cell. (**E**) Cell viability of H62 treatment for 48 h after knocking down STING in B16F10 cells. (**F**) Cell viability of H62 treatment for 48 h after knocking down STING in A375 cells (n = 3).

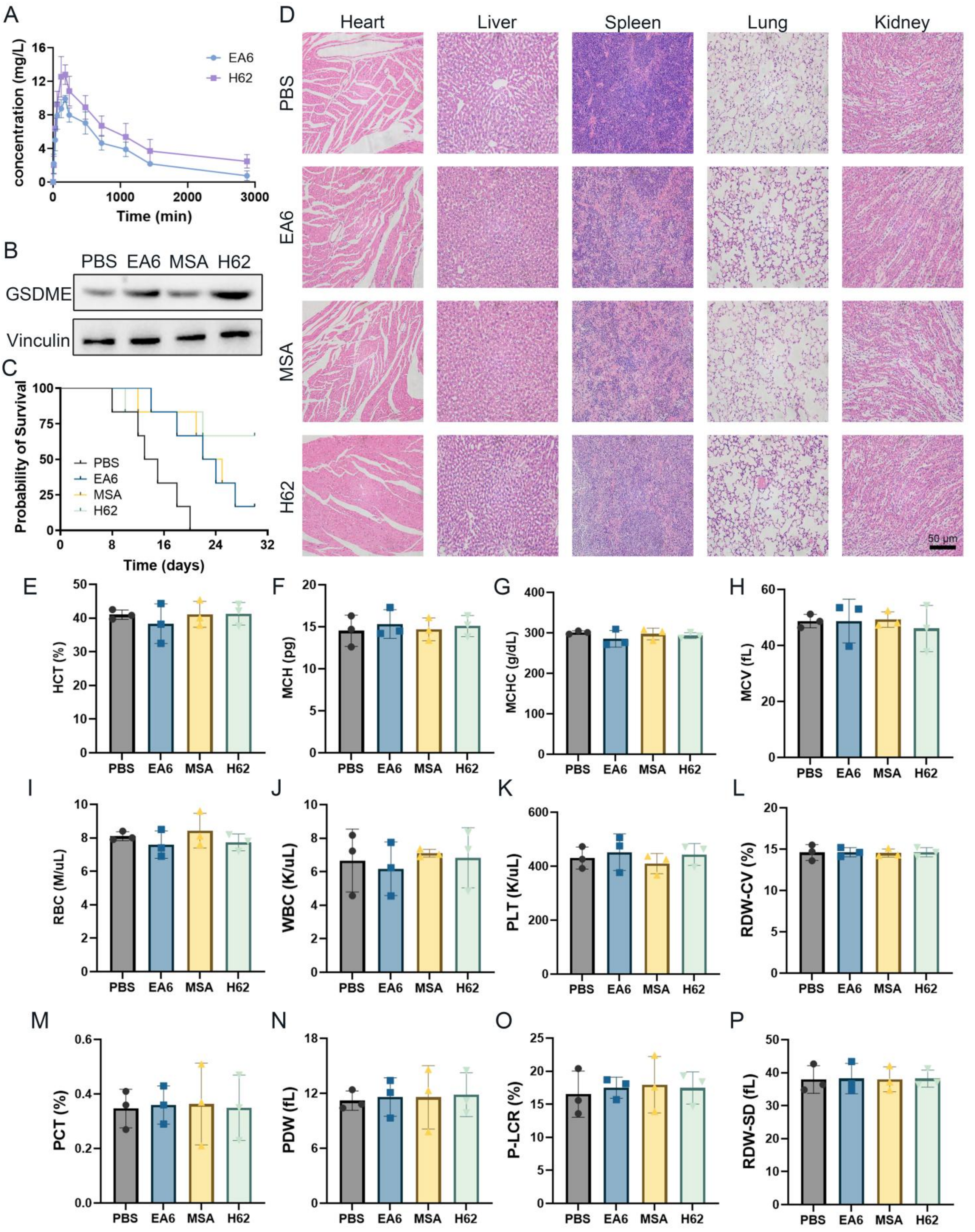

Fig. S13 Evaluation of *in vivo* side effect of different compound treatment groups. (**A**) The concentration-time curves of EA6 and H62 after a single intraperitoneal administration at a dose of 30 mg/kg (n = 3). (**B**) Western blot results of GSDME in tumor tissues from various treatment groups.

(**C**) Survival period of mice receiving different treatments (n = 6). (**D**) The representative H&E staining results of major organs in different compounds treatment groups. (**E-P**) Blood routine results of mice treated with various compounds (n = 3).

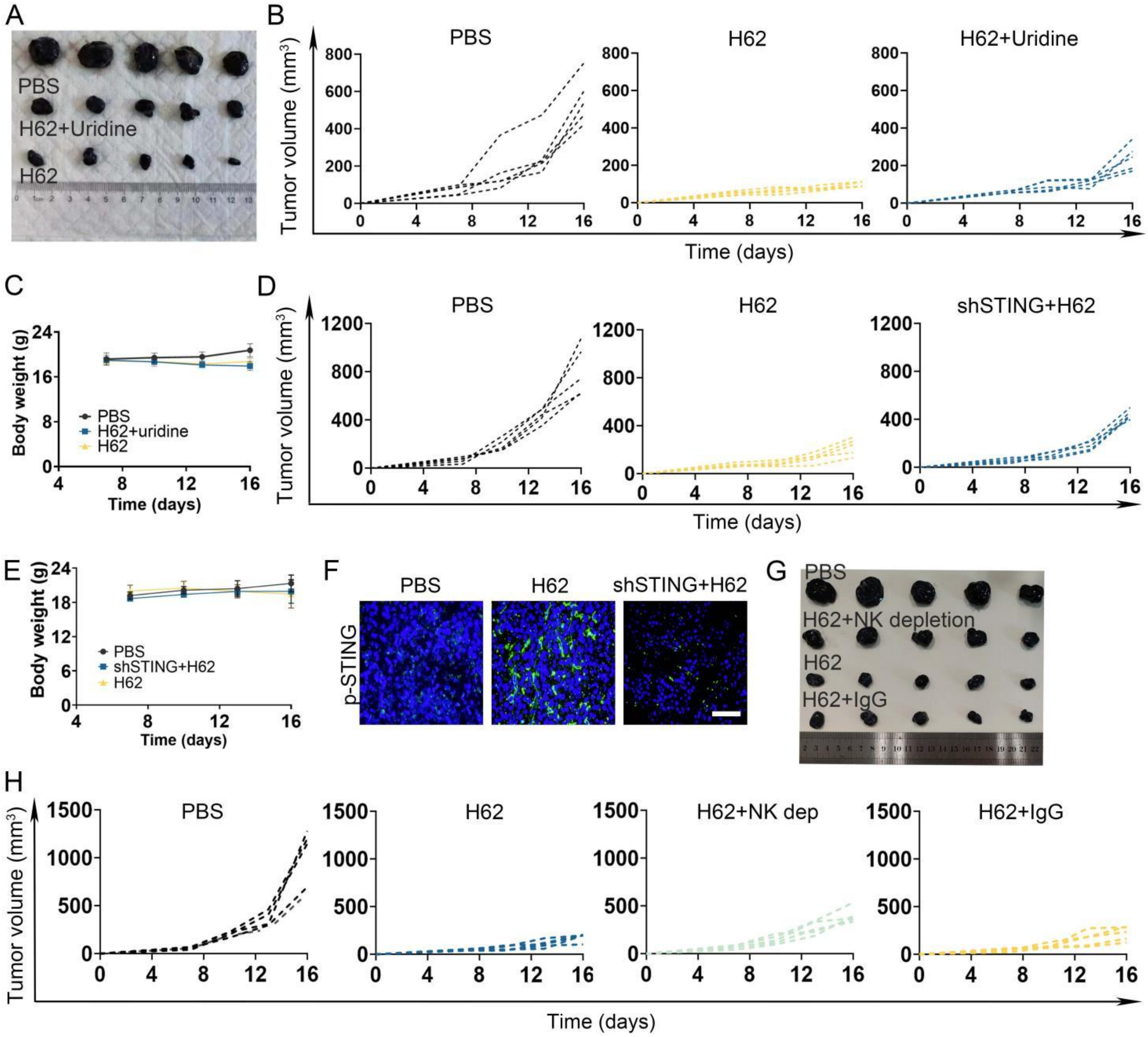

Fig. S14 The antitumor mechanism of H62 *in vivo*. (**A**) The tumor image of different treatment groups (n = 5). (**B**) The individual tumor growth curves of each group mice during uridine rescue experiment (n = 5). (**C**) Body weight of mice in various group during experiment (n = 5). (**D**) The individual tumor growth curves of each group mice in STING knockdown experiment (n = 5). (**E**) Monitoring of mice body weight during the experiment (n = 5). (**F**) p-STING staining of tumor tissues in various treatment groups. Scale bar = 50 μm. (**G**) The image of tumors of different treatment groups (n = 5). (**H**) The individual tumor growth curves of each group mice during NK cell depletion experiment (n = 5).

Table S1. siRNA sequences targeting DHODH.

Target	Sequence (5'-3')
Mouse si#1 sense	GACGGACUGAUCAUCACAA(dT)(dT)
Mouse si#1 antisense	UUGUGAUGAUCAGUCCGUC(dT)(dT)
Mouse si#2 sense	GGCUAGCUGUUCGAGUCAU(dT)(dT)
Mouse si#2 antisense	AUGACUCGAACAGCUAGCC(dT)(dT)
Mouse si#3 sense	GCUGUGGACGGACUCUAUA(dT)(dT)
Mouse si#3 antisense	UAUAGAGUCCGUCCACAGC(dT)(dT)
Human si#1 sense	GGUAUGGAUUUAACAGUCA(dT)(dT)
Human si#1 antisense	UGACUGUUAAAUCCAUACC(dT)(dT)
Human si#2 sense	GAUGUAUGCACUCACCCAA(dT)(dT)
Human si#2 antisense	UUGGGUGAGUGCAUACAUC(dT)(dT)
Human si#3 sense	GUUGAGAUAGGAAGUGUGA(dT)(dT)
Human si#3 antisense	UCACACUUCCUAUCUCAAC(dT)(dT)

Table S2. shRNA sequences targeting STING.

Target	Sequence
Mouse shSTING#1	CCGGATGATTCTACTATCGTCTTATCTCGAGATAAGACGATAGTAGAATCATTTTTTT
Mouse shSTING#2	CCGGCAACATTCGATTCCGAGATATCTCGAGATATCTCGGAATCGAATGTTGTTTTTT
Mouse shSTING#3	CCGGAGAGGTCACCGCTCCAAATATCTCGAGATATTTGGAGCGGTGACCTCTTTTTTT
