# Supplementary material for: An immunometabolic prodrug strategy overcomes DHODH inhibitor resistance in refractory melanoma
Source: J Exp Clin Cancer Res. 2025 Nov 14;44:306. doi: 10.1186/s13046-025-03566-6 (PMC12619403; doi:10.1186/s13046-025-03566-6)

The original images of western blot for

An Immunometabolic Prodrug Strategy Overcomes DHODH Inhibitor Resistance in Refractory Melanoma

The original images of Figure 1A

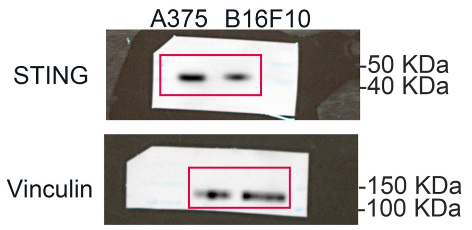

The original images of Figure 1D

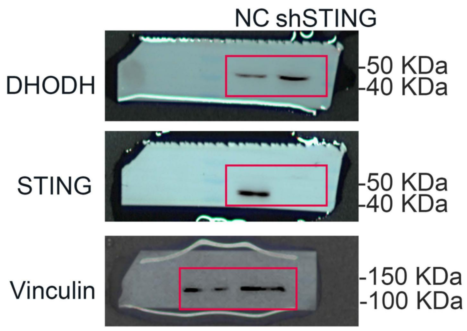

The original images of Figure S1F

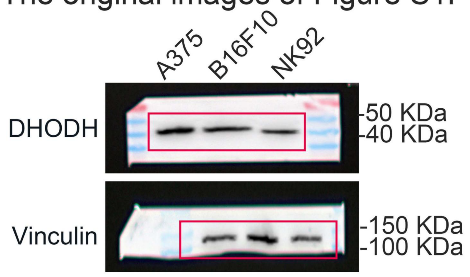

The original images of Figure 3A

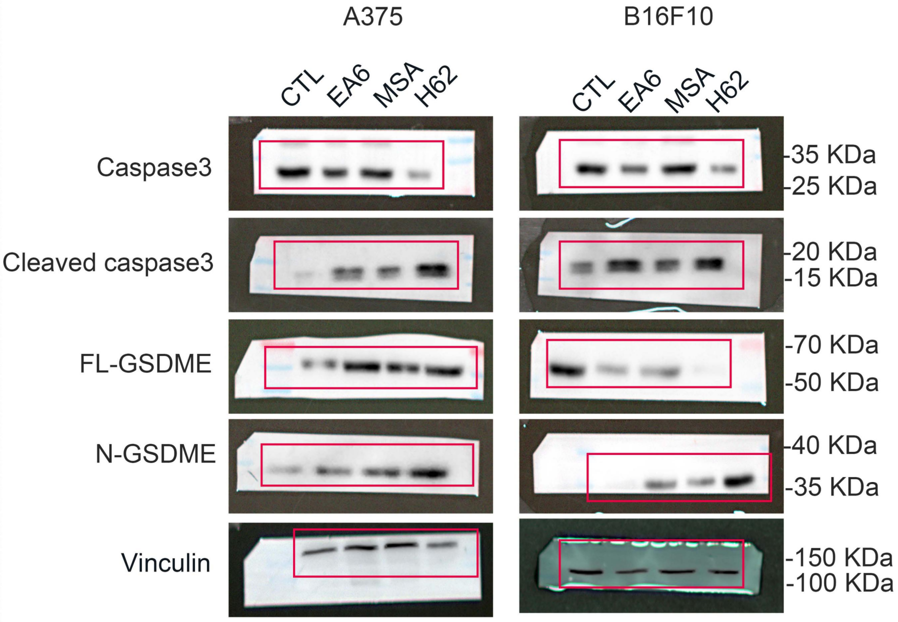

The original images of Figure 3H

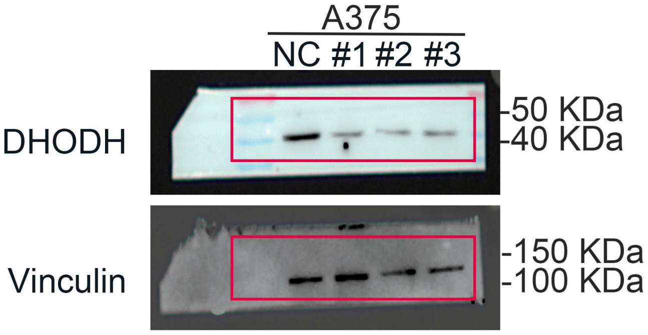

The original images of Figure 3I

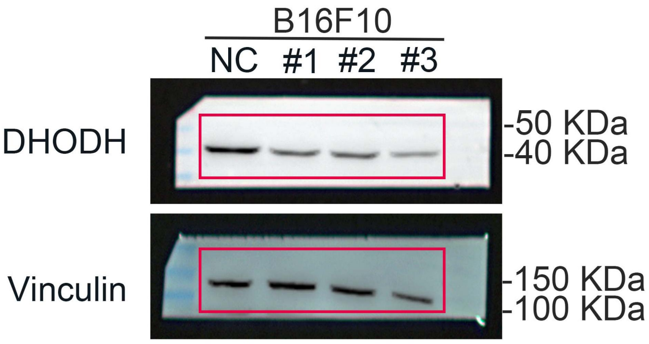

The original images of Figure 3O

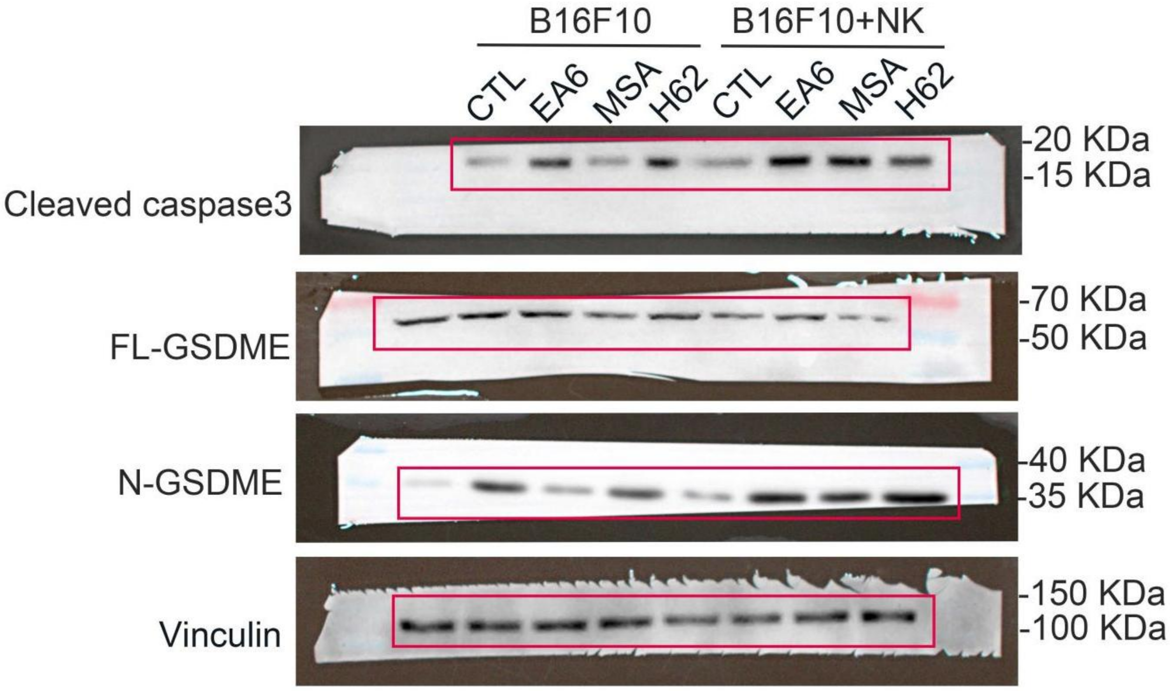

The original images of Figure 4A

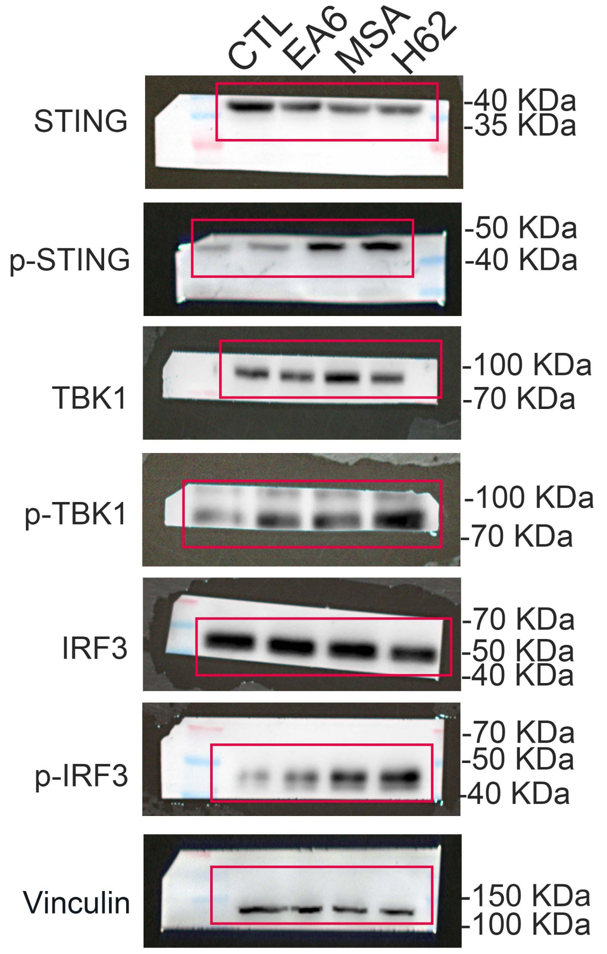

The original images of Figure 4B

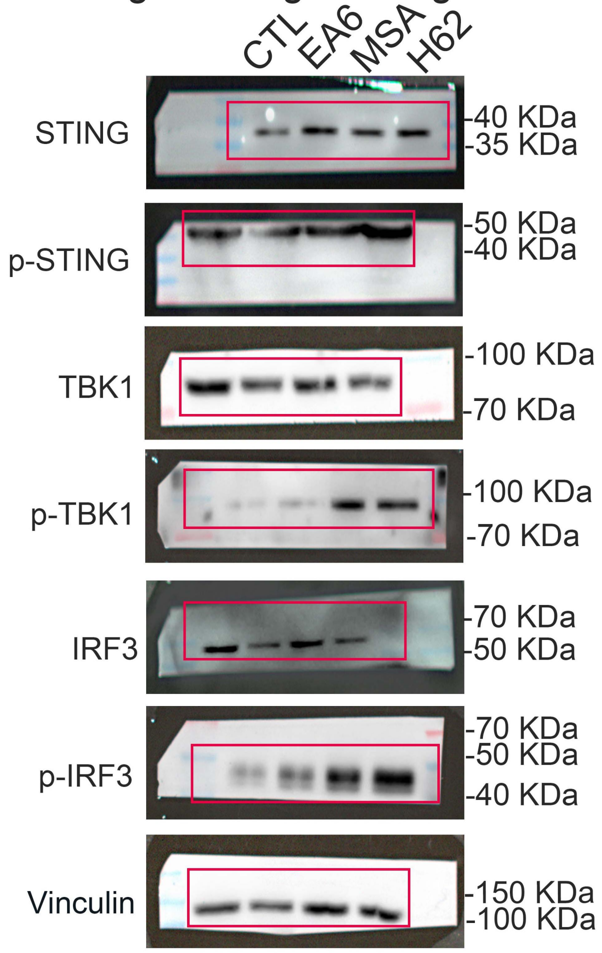

The original images of Figure 4C

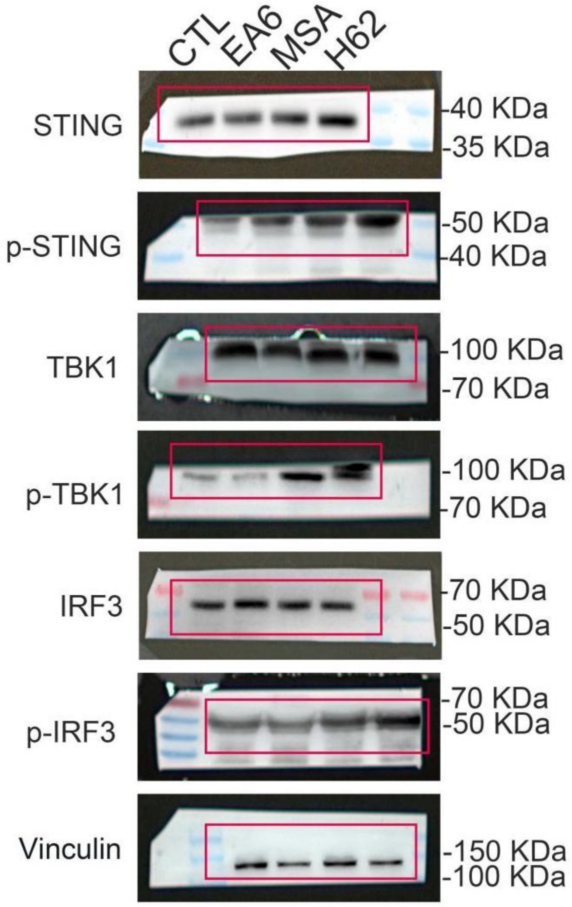

The original images of Figure S4B

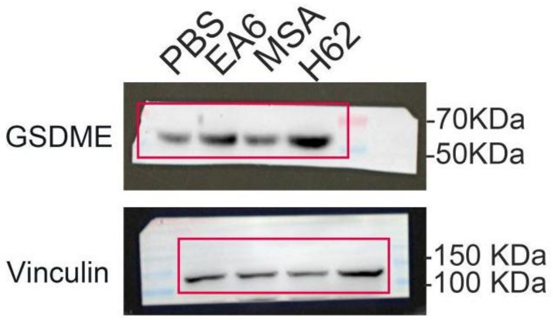

Supplement: Supplementary file 2 — Supplementary Material 2. [file 13046_2025_3566_MOESM2_ESM.pdf]
